# Screening and diagnosis of triple negative breast cancer based on rapid metabolic fingerprinting by conductive polymer spray ionization mass spectrometry and machine learning

**DOI:** 10.3389/fcell.2022.1075810

**Published:** 2022-12-15

**Authors:** Yaoyao Song, Yan Zhang, Songhai Xie, Xiaowei Song

**Affiliations:** ^1^ Department of General Surgery, The Sixth Medical Center of Chinese PLA General Hospital, Beijing, China; ^2^ Department of Burn and Plastic Surgery, The Fourth Medical Center of Chinese PLA General Hospital, Beijing, China; ^3^ Department of Chemistry, Fudan University, Shanghai, China

**Keywords:** ambient ionization mass spectrometry, metabolomics, clinical screening and diagnosis, biomarkers, triple-negative breast cancer

## Abstract

We present the use of conductive spray polymer ionization mass spectrometry (CPSI-MS) combined with machine learning (ML) to rapidly gain the metabolic fingerprint from 1 μl liquid extraction from the biopsied tissue of triple-negative breast cancer (TNBC) in China. The 76 discriminative metabolite markers are verified at the primary carcinoma site and can also be successfully tracked in the serum. The Lasso classifier featured with 15- and 22-metabolites detected by CPSI-MS achieve a sensitivity of 88.8% for rapid serum screening and a specificity of 91.1% for tissue diagnosis, respectively. Finally, the expression levels of their corresponding upstream enzymes and transporters have been initially confirmed. In general, CPSI-MS/ML serves as a cost-effective tool for the rapid screening, diagnosis, and precise characterization for the TNBC metabolism reprogramming in the clinical practice.

## Introduction

Triple-negative breast cancer (TNBC) accounts for 15–20% of breast cancer and ranks the most aggressive and lethal cancer featured as recurrence and distant metastasis (Ahmad, A., 2019). TNBC patient has a poor prognosis in case of metastatic relapse, with a median overall survival (OS) of less than 2 years (Schmid et al., 2020). To date, clinical breast examination (CBE), and radiographic imaging, such as mammography, and magnetic resonance imaging (MRI), are routinely applied in clinical practice for screening, diagnosis, and prognosis assessment of TNBC patients. However, minor molecular changes at the genotype and phenotype basis might be out of surveillance. A highly sensitive and specific molecular screening approach is in urgent demand for early intervention and remarkable improvement in the survival rate.

In past decades, the advent of omics technologies has provided an opportunity to explore large volumes of data at the molecular basis. It may help with a comprehensive understanding of molecular alterations and the underlying mechanisms during cancer development (Xiao et al., 2022; Yang, L., et al., 2020). The molecule-based *in vitro* diagnosis (IVD) has shown promise in the novel diagnostic method development, heterogenic subtype classification, and optimal therapy design (Bianchini, G., et al., 2022). Oncogenic changes may cause metabolic reprogramming of cancer cells to support their uncontrolled growth and help them adapt to the local microenvironment. Metabolomics has been widely accepted in capturing global metabolic changes (Nicholson and Lindon, 2008; Fiehn, O., 2002) and investigating TNBC oncogene initiation (Levine and Puzio-kuter, 2010; Iurlaro, R., et al., 2014), which may provide personalized diagnostic markers (Huang, S., et al., 2016.) and treatment (Debik, J., et al., 2019). Although extensive efforts have been made to identify potential TNBC-associated metabolites from patient blood and tissue (Günther, U.L., 2015; Li, L., et al., 2020; McCartney, A., et al., 2018), metabolomics-based screening and diagnosis have not been put into the frontline of clinical practice yet. From the methodological consideration, it is partially due to the lack of low-cost and high-throughput analytical tools. A successful IVD tool in clinics requires comprehensive considerations from several aspects 1) characteristic markers that are measured with high specificity and sensitivity, 2) suitable biological fluid that is easily accessed, and 3) technical platform with affordable cost, robust test performance, and rapid result feedback (Banerjee, S., 2020).

In recent years, ambient ionization mass spectrometry (AIMS) has gained wide attention in the clinical metabolomics field because of its unique advantages in the direct detection of metabolites and lipids from the biospecimen under the atmospheric condition (Ferreira, C.R., et al., 2016; Feider, C.L., et al., 2019; Narayanan et al., 2020). Compared with the conventionally used liquid or gas chromatography-tandem mass spectrometry (LC-MS/MS, GC-MS) system, AIMS directly acquires hundreds of metabolites and lipids from biological specimens and thus save clinical practitioners lots of efforts spent on labor-intensive sample preparation (Takats, Z., et al., 2017). As one of the representative AIMS methods, conductive polymer spray ionization mass spectrometry (CPSI-MS) (Song, X., et al., 2018.) has been developed and utilized to investigate the metabolic profile from biological fluids. With aid of machine learning technique, features in the salivary metabolic profile can be automatically picked out and used to discriminate the oral squamous carcinoma from the premalignant lesion, and healthy control with high accuracy (Song, X., et al., 2020). These features are selected by the machine learning model from the mathematical point other than the biological interpretable aspect.

CPSI-MS is reported to have wide coverage of metabolite and lipid species with a single test including polar species such as carbohydrates, carboxylates, purines, pyrimidines, polyamines, amino acids and hydrophobic species like fatty acids, acyl carnitines, glycerides, glycerophospholipids, sphingolipids, *etc*. (Li, C., et al., 2021; Yang, X. et al., 2022). This advantage in a wide coverage of multiple species motivates us to explore whether CPSI-MS could precisely gain onco-metabolites located in a certain pathway or involved in certain biological function associated to the TNBC cancer metabolism reprogramming. In this way, the discriminative metabolite markers discovered in serum and tissue can be traceable, and more importantly, biological interpretable.

**FIGURE 1:**
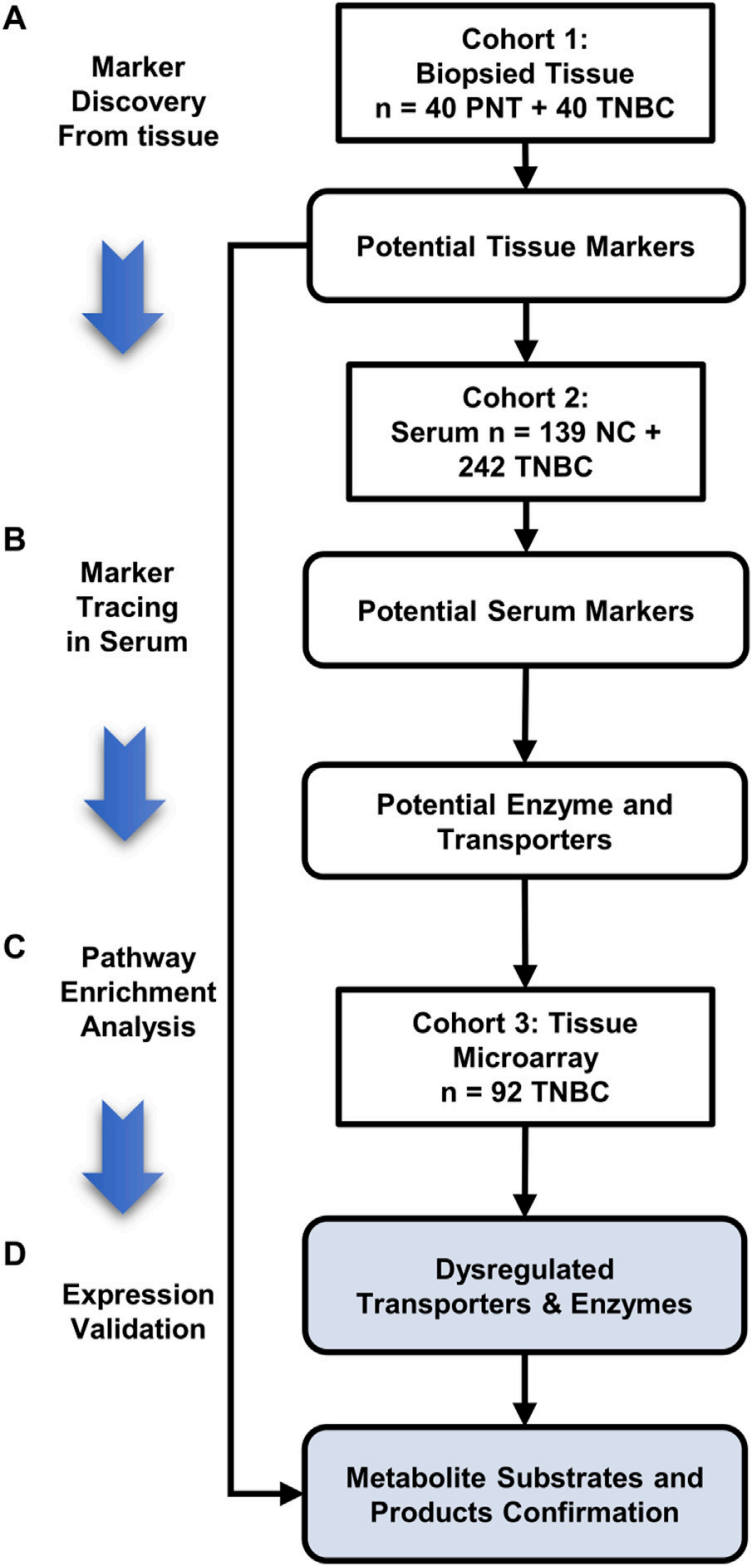
Three stages of this TNBC tissue-serum joint metabolomics workflow. **(A)** Marker discovery from biopsied tissue: 240 sampling points collected from 40 pairs of TNBC and PNT tissues are tested to discover the dysregulated metabolite markers by CPSI-MS and DESI-MSI; **(B)** marker tracing in serum: A cohort composed of 139 HD and 242 TNBC serum is tested by CPSI-MS to trace and cross validate the metabolites discovered in tissue metabolic fingerprinting; **(C)** Pathway enrichment analysis to pick out the potential enzymes and transporters involving in the TNBC tissue/serum metabolite dysregulation; **(D)** Expression validation: Through pathway enrichment analysis, upstream enzymes and transporters that involved metabolite markers regulation are located and validated on the TNBC tissue microarray and immunohistochemistry (IHC). The corresponding metabolite substrates and products are retrospectively examined and confirmed by DESI-MSI.

In this study, we employed CPSI-MS to systematically characterize the distinct onco-metabolites of TNBC, for both serum screening and tissues diagnosis. We followed a workflow that contains four stages in the present study: 1) the *de novo* discovery of the discriminative metabolites in TNBC tissue; 2) track these TNBC-associated metabolites in serum, and construct metabolite-based machine learning models for rapid serum screening and tissue diagnosis respectively; 3) pathway enrichment analysis to locate the potential enzyme and transporter associated with the dysregulated metabolites; 4) expression validation of enzymes or transporters behind these discriminative metabolites on the tissue microarray (Figure 1). In this study, we show that the CPSI-MS serves as not only a diagnostic tool by rapid metabolic fingerprinting but also a powerful way to precisely decode functional metabolites for the TNBC progression and fundamental understanding of its metabolism reprogramming.

## Methods and materials

### Tissue and serum collection

The pairs of TNBC and its adjacent precancerous normal tissue (PNT) were harvested in the department of general surgery, Chinese PLA General Hospital after the tissue biopsy and surgical resection from *n* = 40 stage I-IV TNBC patients during the year of 2018–2022. The serum samples were also collected from the *n* = 242 TNBC patients and *n* = 139 healthy donor (HD) during the same period and places. TNBC cases were confirmed with pathological diagnosis in the department of pathology whereas the HC volunteers are visiting patients who have the negative diagnosis results. The race, ages, and body weight index were strictly matched between the two groups. More details about patients’ clinical demographics were presented in the Supplementary Material (Supplementary Table S1).

### CPSI-MS fingerprinting of TNBC tissue and serum

The methanol-water (1:1, v/v) was used as the solvent that extract metabolites and lipids from biopsied tissue cryosections. For each tissue, three aliquots of solvent droplets were randomly micro-pipetted on different regions and tested by CPSI-MS separately for sake of tissue heterogeneity. A droplet of 5 μl solvent was first spotted and stayed onto a cryosection surface for 30 s. Then it was aspirated back to the micropipette and transferred onto the tip of conductive polymer substrate, which was positioned towards the MS inlet at the distance of 13.0 mm. When the ±4.5 kV direct current high voltage was applied on the conductive polymer tip to trigger the high electric field-induced droplet spray ionization. This process carries the metabolite ions into an LTQ Orbitrap Velos mass spectrometer (Thermo Scientific, San Jose, CA, United States) for the data collection. An untargeted metabolic fingerprinting was conducted under both positive and negative modes within a range of *m/z* 50–1,000. The MS capillary temperature was set at 275°C with the S-lens voltage set at 55 V. The micro-scan number was set at 1 scan which lasted for 200 microseconds as maximum injection time. The data acquisition period for each case lasts for 10 s to collect sufficient data. With respect to the serum fingerprinting, every detail about CPSI-MS is same as that for tissue analysis described above except directly loading the 1 μl serum onto the tip to form a dried spot at the beginning.

The average intensity of each metabolite ion was normalized with the average total ion current (TIC) of each sample. The target discriminative metabolite’s concentration in serum was quantitatively estimated by comparing its normalized intensity with that generated by the commercially available metabolite standard spiked into the serum. Quality control (QC) samples were prepared by pooling equal volumes of serum samples, 20 from TNBC and 20 from healthy donor (HD) group. QC samples were analyzed throughout the run to evaluate the systematic fluctuation. TNBC and HD serum were alternatively arranged for test run with the QC samples evenly inserted into the entire sequence every 30 samples.

### Metabolomics data processing

Batch of raw files were first converted to cdf format using the Xcalibur software (Thermo Fisher Scientific, San Jose, CA, United States). Then the batch of cdf files were imported into MATLAB 2020a for further data preprocessing using the in-built functions and self-programmed scripts. Briefly, each sample’s average mass spectrum was constructed based on 10 continuous scans in the corresponding time window. The metabolite ion’s exact *m/z* value within ±0.005 Da mass tolerance will be defined as a mass bin for peak intensity extraction. Finally, a data matrix composed of peak intensities from all samples was constructed for univariate analysis, multivariate analysis, and machine learning model development. After TIC normalization, the matrix went through natural logarithm transform and then was centered at zero with standard deviation scaled at one, ruling out the magnitude’s biasing influence on the classification modeling.

### Statistical analysis and machine learning

Univariate analysis was first implemented to search for significantly changed metabolite ions among TNBC, and HD groups using Student’s t-test. The *p* values were adjusted with the false discovery rate (FDR) using Benjamini-Hochberg method. An ion was picked out if the fold change was over 2.0 or less than 0.5 (FDR <0.05). For multivariate analysis, SIMCA-P (Umetrics, Umea, Sweden) was used for partial least squares discriminant analysis (PLS-DA) of metabolic profiles. Variables with importance in projection (VIP) larger than 1.0 were considered to make a high contribution in pattern recognition of different groups. Data visualization was carried out using Prism (GraphPad Software, United States) such as heatmap, receiver operating characteristic curve (ROC), scatter plot, volcano graph, *etc*. Lasso regression was employed to develop the machine learning model using the in-built “lasso” function in the MATLAB 2021a (Mathworks, Natick, MA, United States). The 10-manifold cross-validation was carried out by randomly splitting the 240 tissue points or 381 serum samples into a training set and a test set with a ratio of 9:1 for every round of validation.

### Validation of targeted metabolites in tissues by DESI-MSI

For tissue imaging, a commercial 2D DESI system (Prosolia, Indianapolis, United States) was employed in both positive and negative ion scan modes. High voltage of ±5.0 kV was provided by the commercial mass spectrometer and applied onto the sprayer head to generate the electrospray for desorbing and ionizing the components within the cryosection tissue. Methanol-water (7:3, v/v) was used as the spray solvent with the flow rate set at 2.0 μl/min under nebulizer gas pressure of 120 psi. The impact angle between sprayer head and substrate was set at 55°. The height of sprayer tip and the distance from tip to transport tube were all set at 4.5 mm. The MS acquisition was implemented under the same parameter in CPSI-MS experiment described above. For tissue scanning, the raster speed was set at 0.2 mm/s and the width between two scans was 0.2 mm. Massimager (Chemmind Technologies Co., Ltd., Beijing, China) and a self-programmed MATLAB (Mathworks, Natick, MA, United States) script was used for target ion image reconstruction.

### Identification of metabolite markers

The ions of interest in were first searched through HMDB (http://hmdb.ca, accessed on 2022/11/10) and Metlin (https://xcmsonline.scripps.edu, accessed on 2022/11/10) with the mass tolerance set at 5.0 ppm. The type of adduct ions were limited to [M + H]^+^, [M + Na]^+^, [M + K]^+^, [M-H_2_O + H]^+^, [M+2Na-H]^+^, [M+2K-H]^+^, and [M + NH_4_]^+^ under positive mode. The negative ion’s adduct type included [M-H]^-^, [M + Na-2H]^−^, [M + K-2H]^−^, and [M + Cl]^−^. Apart from the exact *m/z* value, the isotope peak distribution was also considered to rule out the less possible formula in the candidate metabolite list. For all the provided compounds, only those candidates with a reported presence in humans were given consideration. For some frequently detected ions such as hypoxanthine, carnitine, spermidine, arginine, *etc.*, we directly give credit to these metabolite assignments because they had been repeatedly identified according to previous studies. For those unknown significantly changed ions, MS/MS experiments were implemented to match the CID fragmentation pattern either with given standards or recorded MS/MS spectra in HMDB and Metlin.

### Bioinformatics analysis

After tentative identification, the metabolites of interest were put into the open-source platform MetaboAnalyst (www.metaboanalyst.ca, accessed on 2022/11/10) to search for these altered metabolic pathways. Pathway enrichment analysis was also implemented by RaMP (Zhang, B., et al., 2018.) cross different databases including Kyoto Encyclopedia of Genes and Genomes (KEGG, www.kegg.jp, accessed on 2022/11/10), and Reactome (www.reactome.org, accessed on 2022/11/10). Enzymes and transporters (only confined to the top 10 enriched pathways) that were significantly different between tumor and normal were picked out in the protein level. After finding these enzymes and transporters, the breast cancer dataset in the cancer genome atlas (TCGA-PAAD) and the GSE15471 datasets in the gene expression omnibus (GEO) (Badea, L., et al., 2008) were reviewed to access their expression differences between tumor and normal in the mRNA level. The statistic process was conducted under an open-source platform, gene expression profiling interactive analysis (GEPIA, cancer-pku.cn, accessed on 2022/11/10) (Tang, Z., et al., 2019).

### Histopathologic evaluation

Apart from the cryosections for CPSI-MS and DESI-MSI analysis, duplicates of parallel sections were processed with standard H&E staining for histopathological evaluation under optical microscopy. Besides, tissue microarray (TMA) test was also carried out for verifying the enzyme and transporter discovered by the pathway enrichment analysis. TMAs containing 43 well preserved formalin-fixed and embedded blocks of 43 patients with paired cancer and paired adjacent tissue were constructed. In our study, each TMA contained 90 spots measured 1.5 mm in diameter. Spot selection was independently checked and selected by two pathologists. Two cores per tumor were selected to eliminate the sample error. Anti-PISD (16401-1-AP,1–300) was obtained from Proteintech company (Shanghai, China). Anti-PDZD11 (ab121210, 1–100) was obtained from Abcam company (Shanghai, China).

## Results

### TNBC tissue fingerprinting

To decipher the features of metabolic profiles in the local microenvironment, we collected biopsied TNBC tissues and precancerous normal tissue (PNT) from *n* = 40 stage I-IV TNBC patients who were prior to treatment. A 15 μm-thick frozen section was used for histopathological evaluation. After hematoxylin and eosin (H&E) staining, slides were annotated by two independent pathologists to define the areas of TNBCs and PNTs. Supplementary Table S1 summarizes clinical characteristics, including gender, age at diagnosis, tumor grade, lymph node involvement, and staging.

**FIGURE 2:**
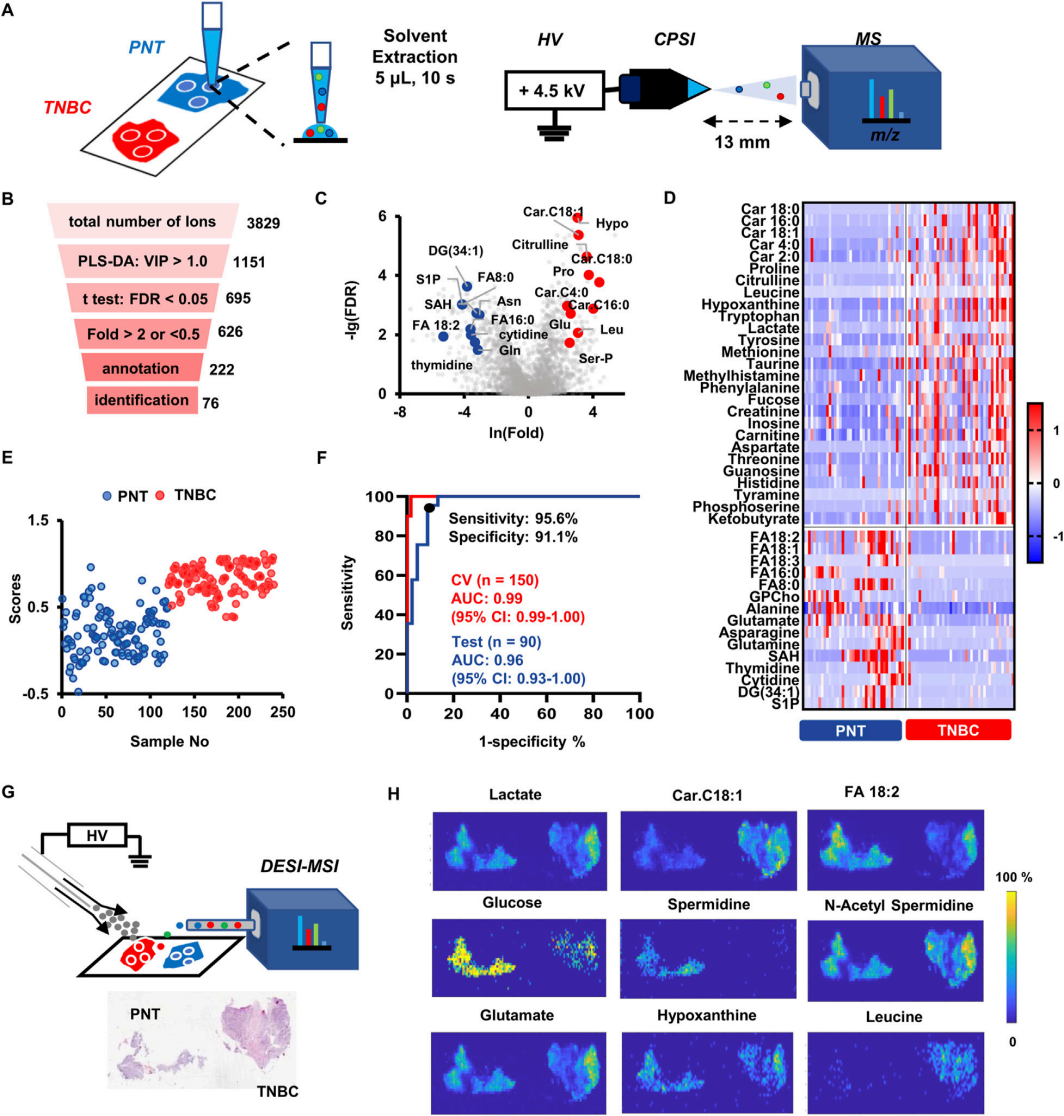
TNBC tissue metabolic fingerprinting, machine learning-based molecular diagnosis and *in situ* validation by DESI-MSI. **(A)** Diagram illustration of the droplet-extraction-based CPSI-MS data collection for the biopsied tissue. Metabolites derived of three different sampling points are collected from each tissue. **(B)** The stepwise statistical pipeline for finally preserving the metabolites that have significant changes in TNBC compared to PNT specimens. **(C)** The volcano plot highlighted those metabolite ions that have significance (FDR <0.05) in fold change over 2.0 or less than 0.5 in TNBCs compared to PNTs. **(D)** Heatmap visualizes the relative expression level of the 40 representative characteristic metabolites in TNBCs and PNTs. **(E)** Plot of all 240 tissue points scores predicted by the Lasso classifier. **(F)** ROC curves for evaluating the Lasso model’s diagnostic performance on training and test set. **(G)** Diagram illustration of the employed DESI-MSI setup for visualizing metabolite’s distribution across a paired TNBC/PNT tissue cryosections. **(H)** Images of representative metabolites that are significantly changed in TNBCs compared to PNTs.

Three different sampling points (spot diameter around 2.0 mm) from each biopsied tissue cryosections were randomly selected for the rapid CPSI-MS fingerprinting. Briefly, a droplet of 5 μl water-methanol (1:1, v/v) solution was first loaded onto the dried tissue cryosection for 10 s’s extraction and then transferred to the tip of conductive polymer tip for following CPSI-MS analysis (Figure 2A). The similar process was also conducted on the later serum metabolomics study. Finally, CPSI-MS simultaneously obtained the relative abundance of 3,829 metabolite ions in the range *m/z* 50–1,000 under both positive and negative modes from each sample. According to the molecular weight distribution ranges of the metabolite species, identified metabolites includes carbohydrates, amino acids, carboxylic acids, polyamines, fatty acids (FA), nucleotides, nucleosides, acylcarnitines (AC), lyso-phosphoglycerolipids, diglyceride (DG), glycerophospholipids (GPL) (Supplementary Figure S1).

Partial least square-discriminant analysis (PLS-DA) was implemented to visualize the case distribution in the feature space from the high-dimension metabolic profile. The PLS-DA score plot showed that sample points from TNBCs and PNTs were well separated into two clusters (Supplementary Figure S2), highlighting the diverse metabolic patterns between tumor and normal tissues. To discover TNBC-specific metabolites, several criteria were adopted to conduct a stepwise variable selection (Figure 2B). First, variable importance on projection (VIP) was employed as the metric, preserving 1,151 ions that were highly contributive to the sample grouping (VIP >1.0). Thereafter, 695 out of 1,151 ions were found to be significantly different between the TNBC and PNT cryosections (FDR <0.05, student’s t-test). For a further selection of the most informative metabolites, we narrowed the scope to 626 ions, with the fold change (FC) greater than 2.0 or less than 0.5 (TNBCs *versus* PNTs). After searching human metabolome databases and Metlin, 222 ions were putatively annotated, among which 76 metabolites were unambiguously identified (Supplementary Table S2). The top 10 up- and down-regulated small metabolites are highlighted by the volcano plot (Figure 2C). Representative small metabolites that significantly changed in TNBCs are shown by the heatmap (Figure 2D). The down-regulated species included asparagine (Asn), sphingosine 1-phosphate (S1P), S-adenosylhomocysteine (SAH), cytidine, thymidine, glutamine (Gln), and fatty acids (*e.g.* FA8:0, FA16:0, FA18:1, FA18:2, FA18:3), whereas the up-regulated species included glutamate (Glu), proline (Pro), leucine (Leu), citrulline, hypoxanthine (Hypo), serine phosphate (Ser-P), and acyl carnitines (*e.g*. carnitine C2:0, C4:0, C16:0, C18:0, C18:1).

Among these metabolites, glutamine has been extensively reported as a conditionally essential nutrient for many cancer cells, which is termed as “glutamine addiction.” Glutamine has been reported to serves as a supplement for energy fueling in TNBC by incorporating into the Krebs cycle through glutaminolysis into glutamate and thereafter deamination into 2-ketoglutarate (Quek, L.E., et al., 2022). There were higher levels of arginine (*m/z* 175.1190, [M + H]^+^) and lysine (*m/z* 147.1128, [M + H]^+^) in TNBCs. These amino acids are precursors for polyamines (Casero, R.A., et al., 2018). While most polyamines (spermine, spermidine, cadaverine) except putrescine (*m/z* 89.1073, [M + H]^+^) were downregulated, their acetylated forms (N-acetyl spermidine, N, N-diacetyl spermidine, N, N-diacetyl spermine, N-acetyl putrescine, and N-acetyl cadaverine) tended to be upregulate in TNBCs. The imbalanced status between acetylation and deacetylation forms may indicate that the strong interaction between polyamines and negatively charged biomolecules (DNA, and RNA) had been greatly decoupled in TNBCs, again suggesting abnormal transcription and translation activities. It is also worth noting that the N, N-dimethyl arginine (*m/z* 203.1503, [M + H]^+^), and N, N, N-trimethyl lysine (*m/z* 189.1597, [M + H]^+^), as the catabolic products of arginine and lysine residues of methylated histones (Hamamoto and Nakamura, 2016), also showed a high abundance in the TNBCs, suggesting an aberrant RNA transcriptional activity in TNBC.

### Machine learning modelling for TNBC tissue diagnosis

To complement with the histopathology-based diagnosis, we introduced the Lasso model to assess the probability of judging whether each CPSI-MS sample is from cancerous or normal tissue. Metabolomics-based modeling was conducted on a training set composed of 150 tissue sample points with histopathological confirmation (*n* = 75 PNT and *n* = 75 TNBC tissue points from *n* = 25 patients). Another *n* = 90 sample points (*n* = 45 PNT and *n* = 45 TNBC tissue points from *n* = 15 patients) were used as the test set for evaluating the performance of the pretrained Lasso model on unseen cases. The 76 metabolites previously discovered by metabolic profiling and *in situ* validation were included as the initial input variable for training. Lasso classifier was employed because it embeds feature selection into the training process by tuning the weight coefficient of each input variable. Only the variable contributive for classification was assigned with non-zero weight.

As a result, the Lasso classifier’s performance reached the highest accuracy on the test set when only preserving 22 out of the 76 variables. Supplementary Table S3 lists selected metabolite markers and corresponding weight coefficients. Given the Lasso prediction score at 0.62 as the threshold, most of the cross-validation samples in TNBC and PNT groups were well distinguished in the score plot (Figure 2E). The confusion matrix showed the classification result given by the optimal Lasso model, which achieved the overall agreement (accuracy) of 93.3% on the test set. The receiver operating characteristic (ROC) curve was introduced to evaluate the optimal diagnostic performance of this Lasso classifier, which achieved on the test set an area under curve (AUC) value of 0.96 (95% confidence interval: 0.93–1.00). Given the cut-off point defined as the highest true positive rate together with the lowest false positive rate on the ROC curve, the best sensitivity and specificity of the diagnostic performance were 95.6% and 91.1%, respectively (Figure 2F). These results suggest that TNBC-specific metabolomics profiles exhibit excellent performance in distinguishing tumor tissues from normal tissues. Hence, the CPSI-MS data acquisition combined with the 22-metabolite Lasso model serve as a potential IVD strategy complementary with tissue biopsy and molecular pathology.

### DESI-MSI validation of TNBC-associated metabolites

Desorption electrospray ionization mass spectrometry imaging (DESI-MSI) was employed in complement with CPSI-MS for validation of various metabolites distribution across the TNBC and PNT regions. The TNBC cryosection paired with its PNT regions were prepared for study cases (Figure 2G). We successfully visualized and matched the spatial distribution of the CPSI-MS-identified 76 metabolites across the TNBC and the PNT regions (representative images shown in Figure 2H). DESI-MSI indicated a dysregulation in energy metabolism. The decrease of glucose (*m/z* 219.0265, [M + K]^+^) in TNBCs and increase of lactate (*m/z* 89.0242, [M-H]^-^) in PNTs is consistent with the Warburg effect (Vander Heiden, M.G., et al., 2009). As for other Krebs cycle-related metabolites, their relative abundance varied across various tissue regions with inconsistent trends. The energy fueling was also indicated by increased production of acylcarnitine (*e.g.*, carnitine C2:0, carnitine 4:0, and carnitine C18:1) and overconsumption of free fatty acids (*e*.*g.*, linoleic acid, arachidonic acid, caprylic acid, and oleic acid) *via* β-oxidation.

### TNBC-associated metabolites tracking in serum

**FIGURE 3:**
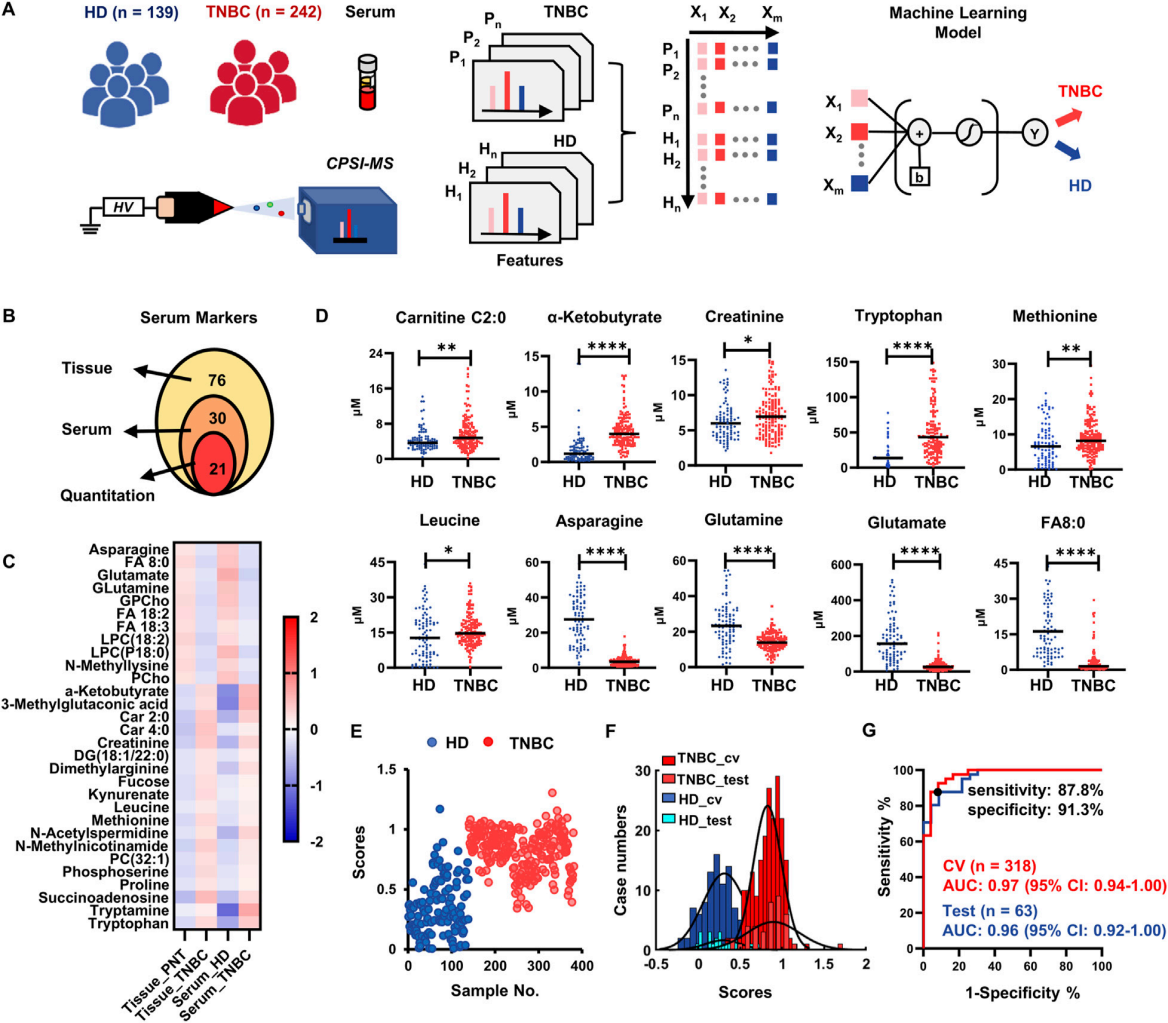
Results of serum validation for metabolite markers discovered in tissue. **(A)** Diagram of recruited cohort of healthy donor (HD), TNBC patients, CPSI-MS screening for serum metabolomics, and machine learning process; **(B)** Venn graphs display the number of metabolites that are finally selected as serum screening markers and used for quantitative estimation; **(C)** Heatmap visualization indicate the consistent tendencies of these characteristic metabolites in tissue and serum; **(D)** The quantitative comparison of representative 10 metabolite markers in serum; **(E)** The scores predicted by Lasso classifier on the 381 TNBC and HD serum samples; **(F)** Histograms display the distribution of HD and TNBC samples in the training and test sets; **(G)** ROC curve for assessing the performances of the metabolite markers-based Lasso model for TNBC screening. *p < 0.05, **p < 0.01, ***p < 0.001, ****p < 0.0001.

To enhance the diagnostic value of the putative TNBC-specific metabolic markers, we conducted tissue-serum joint analysis and inter-specimen cross-validation, followed by untargeted metabolomics analysis. First, we carried out independent serum metabolomics investigation by CPSI-MS analysis under full scan modes ranging in *m/z* 50–1,000. The study prospectively recruited *n* = 242 stage I-IV TNBC patients and *n* = 139 healthy donors (HD) (Figure 3A).

Using the stepwise statistical screening as described above, the serum analysis identified 76 differentially enriched metabolites in the sera of TNBC patients compared with that in the HD group (student’s t-test, FDR <0.05, Supplementary Table S4). Then, a joint analysis was conducted to elucidate the differential metabolomic molecules co-existing in the tumor tissues and sera. The relative abundances of the 76 target metabolites that were significantly changed in the TNBC tissues were cross interrogated in metabolomic profiling of sera. There were 30 metabolites showing the same trends of fold changes in serum compared with those in the tissue metabolomics (FC > 2 or <0.5) (Figures 3B,C; Supplementary Table S5). There were 21 (70%) out of the 30 metabolites which had the commercially available standards which we utilized to implement quantitative estimation and confirmed 18 metabolites (Supplementary Table S6). Among them, 10 upregulated and 8 downregulated metabolites were identified in the serum of TNBC patients compared with those in the HD group (*p* < 0.05, representative 10 metabolites are shown in Figure 3D; the rest was shown in Supplementary Figure S3).

We also used these 18 small metabolites as the initial input variables to develop another Lasso classifier for the purpose of rapid TNBC screening using an additional cohort of *n* = 381 TNBC cases. Through 5-fold cross-validation, the Lasso classifier achieved an average of 89.2% accuracy on the training set (*n* = 318) *versus* 88.9% accuracy on the test set (*n* = 63). Excluding metabolites assigned with zero weights, there were 15 metabolites that were conserved in the serum metabolite classifier panel (Supplementary Table S7). As shown in the Lasso score plot for all 381 cases, most serum samples from the TNBC group could be differentiated from the HDs serum samples (Figure 3E). The histogram plot of the Lasso scores also revealed that the training and test sets shared the close normal distributions (Figure 3F), indicating that the Lasso classifier have the robust prediction ability on those unseen cases. The 15-metabolite Lasso classifier could reach an AUC of 0.96 (95% CI: 0.92–1.00) on the test set, with the 85.4% of sensitivity and 91.3% of specificity at the best cut-off point on the ROC curve (Figure 3G), suggesting the potential clinical value for rapid serum screening.

### Expression validation of associated enzymes and transporters

**FIGURE 4:**
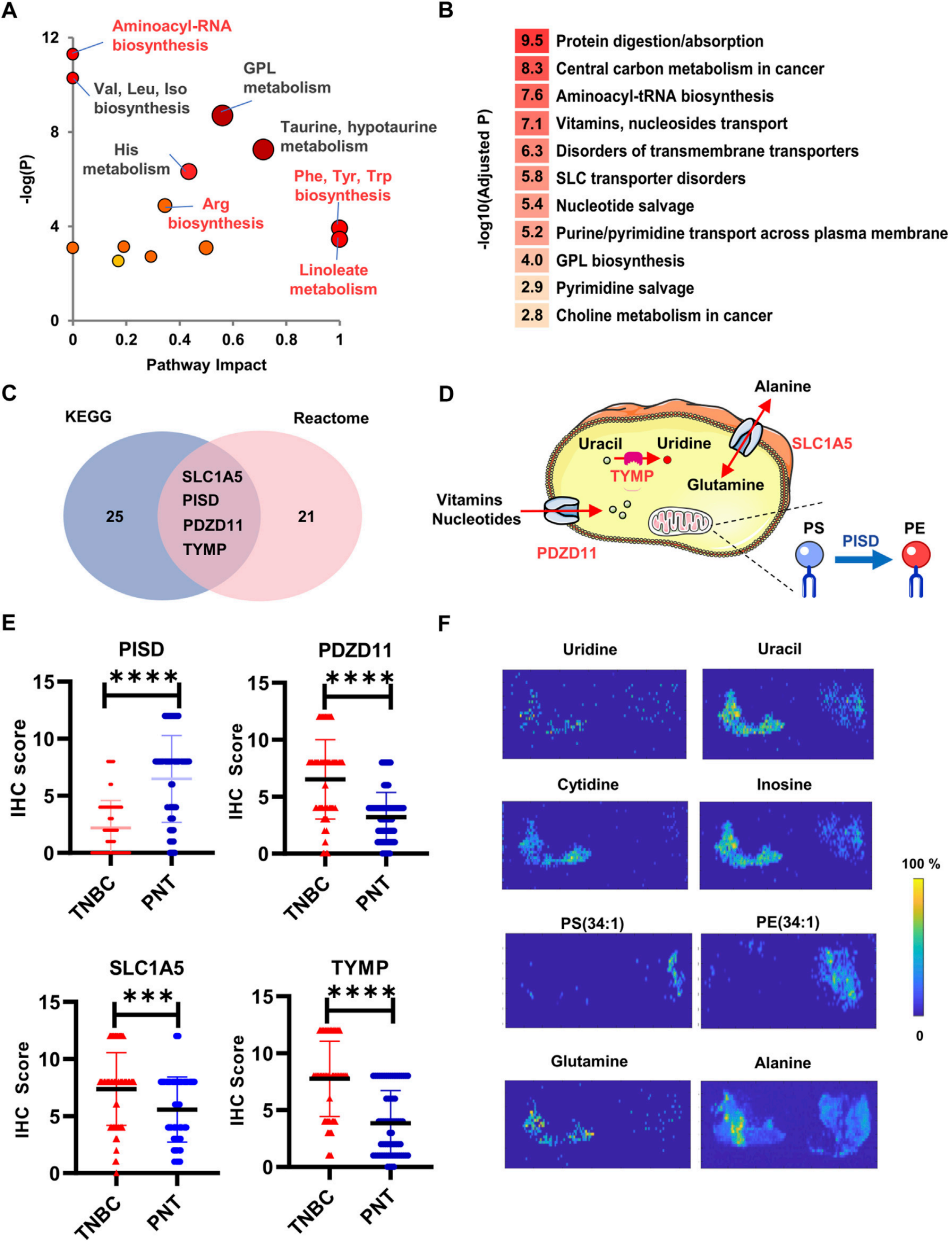
Bioinformatic analysis and expression validation for the dysregulated enzymes and transporters. **(A)** The enriched metabolic pathways by searching in the Metaboanalyst; **(B)** Rank of influenced biological functions that enzymes, and transporters are involved; **(C)** The enriched enzymes and transporters by searching in KEGG and Reactome; **(D)** Diagram illustrates the subcellular locations and functions of the PDZD, PISD, TYMP, and SLC1A5; **(E)** Box plots of PISD, PDZD11, TYMP, and SLC1A5 across TNBC and PNT regions; **(F)** Distribution across the PNT (left) and TNBC (right) for metabolite substrates and products corresponding to TYMP (Uridine, Uracil), PDZD11 (Cytidine, Inosine), PISD (PS(34:1) and PE (34:1)), and SLC1A5 (Glutamine, Alanine). ****p < 0.0001.

To further determine biological consequence and metabolic network using the 76 metabolites identified in TNBC tissues by CPSI-MS and DESI-MSI, pathway enrichment analysis (PEA) was conducted on MetaboAnalyst (Pang, Z., et al., 2021) to predict the relevant metabolic pathways during TNBC development and progression. We found that these differentially enriched metabolites in TNBC tissues were involved in distinctive metabolic pathways (Figure 4A). Specifically, taurine and hypotaurine metabolism (*e.g.,* cysteate, taurine, and hypotaurine), glycerophospholipid metabolism (*e.g.,* PE, PC, lysoPC, DG, PCho, PS, GPCho, GPEA, and G3P), histidine metabolism (*e.g*., glutamate, urocanate, histidine, methylhistamine, histamine, and aspartate), glutamate and glutamine metabolism (*e.g*., glutamate, glutamine, and ketoglutarate), linoleate metabolism (linoleate and phosphatidylcholine), cysteine and methionine metabolism (*e.g*., serine, methionine, cysteate, and phosphoserine), aromatic amino acid biosynthesis (*e.g*., phenylalanine and tyrosine), and arginine-related metabolites (*e.g*., arginine, citrulline, aspartate, and ornithine) were commonly enriched both in TNBC tissues and serums (Supplementary Figure S4; Supplementary Tables S8,S9).

Thereafter, the PEA was further conducted by searching all TNBC-associated metabolite markers and influenced functions in Kyoto Encyclopedia of Genes and Genomes (KEGG, www.kegg.jp), and Reactome (www.reactome.org). The most influenced biological functions that enzymes, and transporters involved the protein digestion and transport of various nutrients (Figure 4B). Totally, there were 56 enzymes or proteins predicated to have different extents of suppression or activation. Among these, four metabolic enzymes and transporters ranked at the top of the candidate list highlighted with statistical significances (*p* < 0.05, Figure 4C). The phosphatidylserine decarboxylase (PISD) was down-regulated whereas PDZ domain-containing protein 11 (PDZD11), thymidine phosphorylase (TYMP), and solute carrier family 1 member 5 (SLC1A5) were upregulated. PDZD11 and SLC1A5 are mainly involved in the transport of water-soluble amino acids, vitamins, free purines and pyrimidines, nucleotides, and nucleosides (Scalise, M., et al., 2018; Zhou, Y., et al., 2020). PISD is mainly responsible for the conversion of PS to phosphatidylethanolamine (PE) (Thomas, H.E., et al., 2018; Ma, Y., et al., 2020), which has been reported to play a tumor-suppressing role (Humphries, B.A., et al., 2020). TYMP catalyzes thymidine/deoxyuridine to thymine/uracil, respectively (Goto, T., et al., 2012) (Figure 4D). It was previously reported that TYMP-dependent thymidine catabolism contributes to cancer cells’ survival in low nutrient conditions (Toi, M., et al., 2005). Consistently, immunohistochemistry (IHC) analysis using tissue microarray that included *n* = 92 TNBC primary cancer specimens confirmed this result, showing that the expression of PISD was decreased, whereas the expressions of SLC1A5, TYMP, and PDZD11 were significantly increased in TNBC tissues compared with PNTs (Figure 4E).

We also conducted a retrospective DESI-MSI investigation on the expression levels of metabolite substrates and products specific to the four enzymes or transporters above. From both CPSI-MS and DESI-MSI analysis, we observed that glutamine and alanine were significantly lower in TNBC. SLC1A5 mainly mediates the transmembrane exchange of extracellular glutamine for cytosolic alanine. Therefore, this result supported the up-regulation of SLC1A5 in the TNBC cell to raise the turnover and utilization of glutamine and alanine. PISD catalyzes the conversion of PS into PE. Taking PS (34:1) and PE (34:1) as an example, DESI-MSI showed that the relative abundance of PE, and PS were enriched in TNBC regions. Besides, the lower abundances of uridine, uracil, cytidine, and inosine in TNBA region may indicate the over-consumption and utilization of the nucleotides (Figure 4F), further confirming the increased expression of PDZD11 and TYMP.

## Discussion

CPSI-MS has been proved to be a promising technique to acquire the fingerprinting about metabolites and lipids within a few seconds time scale, which only consumes a trace amount of biofluid (≤1 μl) or biotissue (<1 mg). In this study, only one working day was required to complete collecting 40 pairs of biopsied TNBC tissues and 381 serum samples presented in this work. The speed, accuracy, sensitivity, and selectivity of CPSI-MS/machine learning make this approach highly advantageous in clinics for high-throughput, large-scale IVD from a trace amount of biospecimen.

**FIGURE 5:**
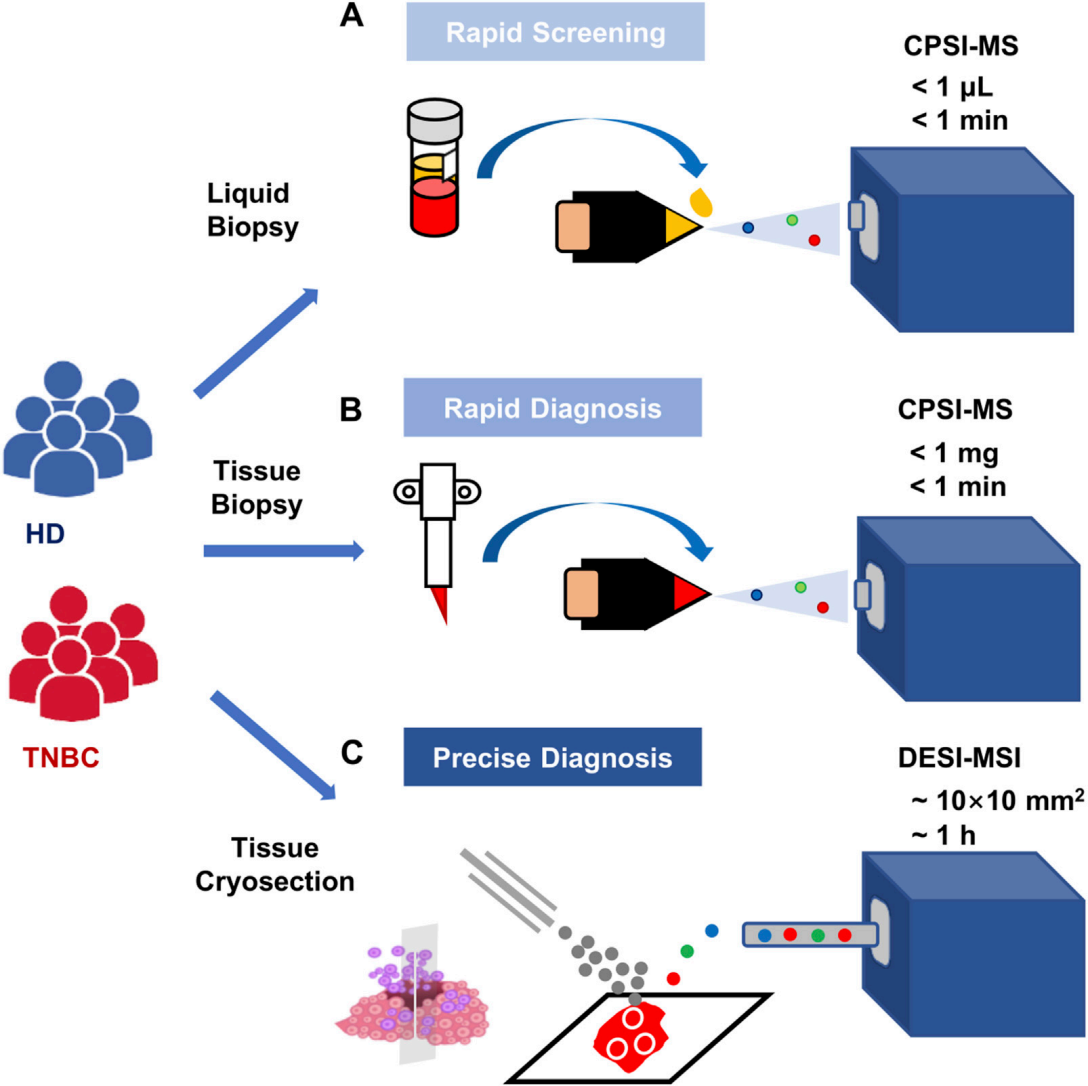
Recommended scenarios for CPSI-MS in clinical application for TNBC screening and diagnosis. **(A)** The high-risk population screening conducted by CPSI-MS only consumes 1 μL serum collected from each clinical recipient and takes less than 1 min to complete metabolic profile data acquisition; **(B)** the metabolic profiling and rapid TNBC diagnosis conducted by CPSI-MS only consumes trace amount of biopsied tissue (less than 1 mg) from these highly suspicious patients; **(C)** more precise diagnosis and spatially resolved metabolic profiling is conducted by DESI-MSI accompanied with molecular pathology on these patients who receive surgical resection of the TNBC tissue.

CPSI-MS and DESI-MSI complement each other for different phases of clinical TNBC examination according to their principles and technical characteristics. CPSI-MS only consumes a trace amount of biofluid (1 μl) or biotissue (<10 mg) for rapid metabolic profiling within a few seconds, hence is suitable for high-throughput and rapid screening. DESI-MSI enables a more in-depth analysis of spatially resolved metabolomics. It does not require tissue fixation, and it has a relatively high spatial resolution (∼100 μm). Hence, we proposed the combination of CPSI and DESI-MSI to complement with each other used in the different stages of clinical scenarios (Figure 5).

One individual’s serum metabolic fingerprint only reflects the general metabolism, which may be influenced by multiple factors such as personal physiological condition, cancer progression, or external perturbation such as diet, smoking, surgery therapy, or drug treatment. Under this situation, a single metabolite marker presented in the serum metabolic fingerprint may have poorer indicative performance. Combining a panel of characteristic metabolites helps to reduce the false negative rate and increase the chance of positive detection. Nonetheless, rapid serum metabolic fingerprinting is recommended for serving the TNBC screening among the susceptible female population. In comparison, the tissue metabolic fingerprint provides a relatively reliable and specific indication of the existence of the carcinoma region. As the downstream molecular products, the cancer tissue’s metabolic fingerprint contains these dysregulated metabolite markers that have a strong connection with upstream oncogenes. Therefore, the metabolite markers deciphered from the tissue metabolic fingerprint show more convincing proof of TNBC occurrence, advancing, and understanding its distinctive metabolism reprogramming strategy compared to the normal tissue cells. Additionally, the chemotherapy or surgical removal of the TNBC tissue may help to alleviate the metabolism burden imposed by cancer, the levels of TNBC metabolite markers in tissue or serum will also be tuned back to a certain extent. Thus, the metabolic fingerprint may also have the potential for prognosis and individual surveillance in the clinic.

Previously, the conventional AIMS-based molecular diagnosis heavily relies on machine learning model to pick out the best combination of the MS peaks as input features for disease prediction. These peaks are selected from the point of mathematical significance other than the biological basis. In this study, CPSI-MS yields a more comprehensive coverage of varied small metabolite species along with lipids. Several functional metabolite markers were successfully deciphered to be associated with TNBC progression and traceable both at the tissue and serum levels. The further expression and function validation about upstream enzymes and transporters gave the discovered metabolites a more valid support as the possible markers.

**FIGURE 6:**
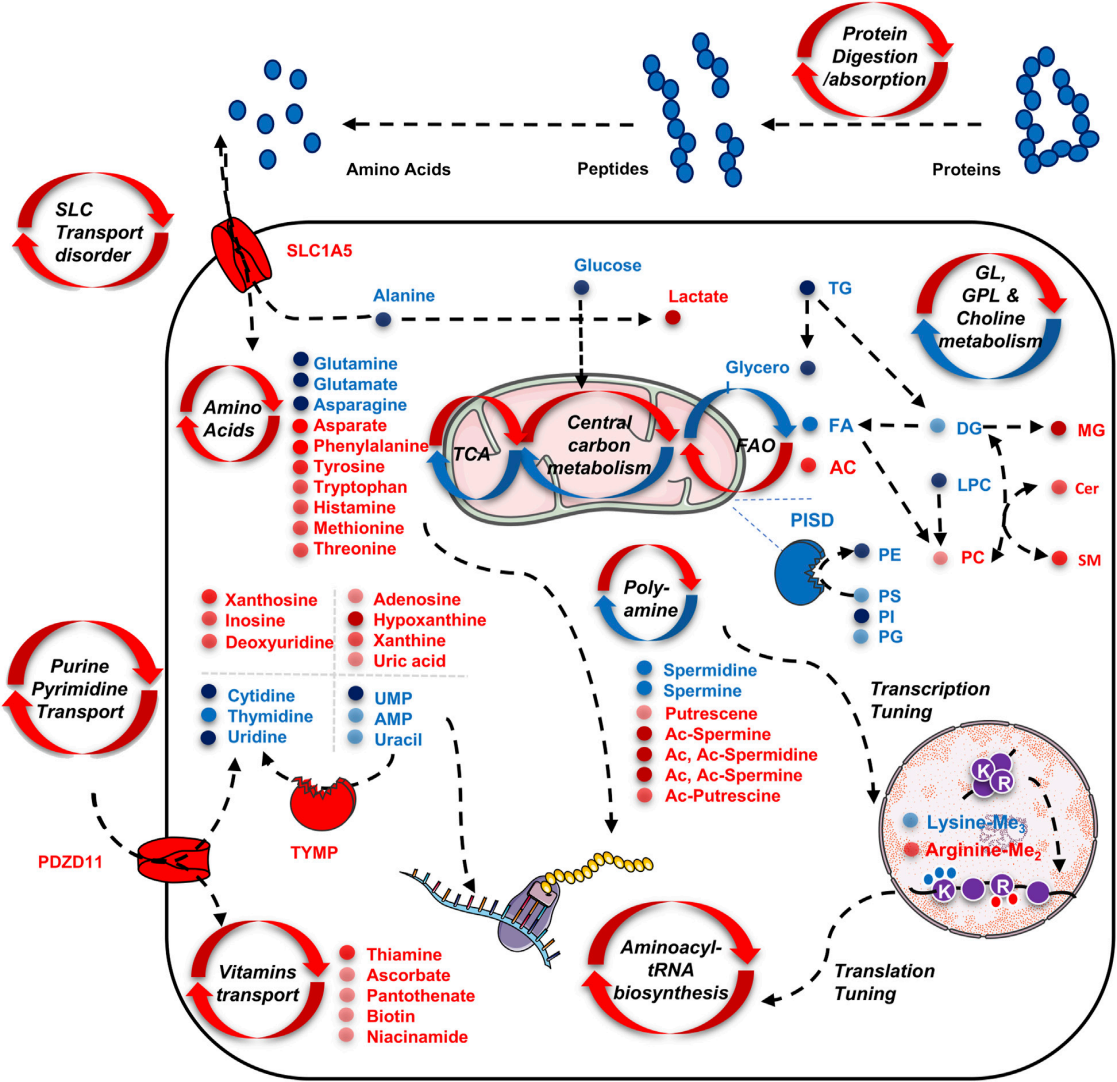
Schematic illustration of the up-regulated and down-regulated metabolites as well as their involved metabolism pathways for adapting the tumor cell’s proliferation and transcription regulation. All metabolites presented in the scheme are discovered by serum and tissue untargeted metabolomics analysis by the CPSI-MS.

Through this study, we summarized and draw a scheme to illustrate the featured TNBC metabolism (Figure 6): 1) active nutrient scavenging and biomass transportation. This pattern was characterized by enriched pathways that highlight functional changes in protein digestion and uptake, SLC transporter disorder, nucleotide and pyrimidine salvage, and vitamin transport. This is supported by elevated expression of SLC1A5 and PDZD11, as well as their downstream changes in amino acids (e.g. glutamine, alanine), nucleosides (*e.g.*, guanosine, inosine, xanthosine), nucleotides (AMP, UMP, and uracil), purines (adenine, hypoxanthine, xanthine, and uric acid), pyrimidines (cytidine, thymidine, and uridine); 2) excessive energy fueling through anaerobic glycolysis and fatty acid oxidation (FAO), as indicated by the down-regulation of glucose, FA, and upregulation of lactate and AC; 3) construction of bilayer membrane components to support cancer cell replication, characterized by the downregulation of PISD and its downstream products PC, PE, PS, and SM, as well as the massive usage of the polar head groups (*e.g,.* GPCho, GPEA, and G3P); 4) abnormal DNA replication, transcription, and RNA translation, indicated by the upregulation of various amino acids, purines, pyrimidines, and the dysregulation of dimethyl arginine and trimethyl lysine, as the breakdown products from histones (Di Lorenzo and Bedford, 2011); 5) aberrant signaling regulation, indicated by increased ratio of acetylated and deacetylated forms of polyamines, as well as decreased DG and increased ceramides, which are related to cell proliferation (Ogretmen, B., 2018; Casero, R.A., et al., 2018).

Rapid CPSI-MS fingerprinting conveniently translates the functional metabolite information from trace tissue and serum for TNBC prediction. The functional metabolite markers-based machine learning model was developed to gain insight into the spatially resolved metabolism status during the tissue biopsy and assist the pathological diagnosis. Our systematic investigation confirmed that the CPSI-MS/ML serves as is a robust approach for rapid screening, diagnosis and precise metabolic characterization for the TNBC.

## Data Availability

The original contributions presented in the study are included in the article/Supplementary Material, further inquiries can be directed to the corresponding author.

## References

[B1] Ahmad A. (2019). Breast cancer statistics: Recent trends. Adv. Exp. Med. Biol. 1152, 1–7. 10.1007/978-3-030-20301-6_1 31456176

[B2] Badea L. Herlea V. Dima S. O. Dumitrascu T. Popescu I. (2008). Combined gene expression analysis of whole-tissue and microdissected pancreatic ductal adenocarcinoma identifies genes specifically overexpressed in tumor epithelia. Hepatogastroenterology. 55 (88), 2016–2027. 19260470

[B3] Banerjee S. (2020). Empowering clinical diagnostics with mass spectrometry. ACS omega 5 (5), 2041–2048. 10.1021/acsomega.9b03764 32064364 PMC7016904

[B4] Bianchini G. De Angelis C. Licata L. Gianni L. (2022). Treatment landscape of triple-negative breast cancer—expanded options, evolving needs. Nat. Rev. Clin. Oncol. 19 (2), 91–113. 10.1038/s41571-021-00565-2 34754128

[B5] Casero R. A. Murray Stewart T. Pegg A. E. (2018). Polyamine metabolism and cancer: Treatments, challenges and opportunities. Nat. Rev. Cancer 18 (11), 681–695. 10.1038/s41568-018-0050-3 30181570 PMC6487480

[B6] Debik J. Euceda L. R. Lundgren S. Gythfeldt H. V. D. L. Garred Ø. Borgen E. (2019). Assessing treatment response and prognosis by serum and tissue metabolomics in breast cancer patients. J. Proteome Res. 18 (10), 3649–3660. 10.1021/acs.jproteome.9b00316 31483662

[B7] Di Lorenzo A. Bedford M. T. (2011). Histone arginine methylation. FEBS Lett. 585 (13), 2024–2031. 10.1016/j.febslet.2010.11.010 21074527 PMC3409563

[B8] Feider C. L. Krieger A. DeHoog R. J. Eberlin L. S. (2019). Ambient ionization mass spectrometry: Recent developments and applications. Anal. Chem. 91 (7), 4266–4290. 10.1021/acs.analchem.9b00807 30790515 PMC7444024

[B9] Ferreira C. R. Yannell K. E. Jarmusch A. K. Pirro V. Ouyang Z. Cooks R. G. (2016). Ambient ionization mass spectrometry for point-of-care diagnostics and other clinical measurements. Clin. Chem. 62 (1), 99–110. 10.1373/clinchem.2014.237164 26467505 PMC6367930

[B10] Fiehn O. (2002). Metabolomics—The link between genotypes and phenotypes. Plant Mol. Biol. 48, 155–171. 10.1023/a:1013713905833 11860207

[B11] Goto T. Shinmura K. Yokomizo K. Sakuraba K. Kitamura Y. Shirahata A. (2012). Expression levels of thymidylate synthase, dihydropyrimidine dehydrogenase, and thymidine phosphorylase in patients with colorectal cancer. Anticancer Res. 32 (5), 1757–1762. 22593457

[B12] Günther U. L. (2015). Metabolomics biomarkers for breast cancer. Pathobiology. 82 (3-4), 153–165. 10.1159/000430844 26330356

[B13] Hamamoto R. Nakamura Y. (2016). Dysregulation of protein methyltransferases in human cancer: An emerging target class for anticancer therapy. Cancer Sci. 107 (4), 377–384. 10.1111/cas.12884 26751963 PMC4832871

[B14] Huang S. Chong N. Lewis N. E. Jia W. Xie G. Garmire L. X. (2016). Novel personalized pathway-based metabolomics models reveal key metabolic pathways for breast cancer diagnosis. Genome Med. 8 (1), 34–14. 10.1186/s13073-016-0289-9 27036109 PMC4818393

[B15] Humphries B. A. Cutter A. C. Buschhaus J. M. Chen Y. C. Qyli T. Palagama D. S. (2020). Enhanced mitochondrial fission suppresses signaling and metastasis in triple-negative breast cancer. Breast Cancer Res. 22 (1), 60–18. 10.1186/s13058-020-01301-x 32503622 PMC7275541

[B16] Iurlaro R. León-Annicchiarico C. L. Muñoz-Pinedo C. (2014). Regulation of cancer metabolism by oncogenes and tumor suppressors. Methods Enzymol. 542, 59–80. 10.1016/B978-0-12-416618-9.00003-0 24862260

[B17] Levine A. J. Puzio-Kuter A. M. (2010). The control of the metabolic switch in cancers by oncogenes and tumor suppressor genes. Science 330, 1340–1344. 10.1126/science.1193494 21127244

[B18] Li C. Li K. Xu X. Qi W. Hu X. Jin P. (2021). A pilot study for colorectal carcinoma screening by instant metabolomic profiles using conductive polymer spray ionization mass spectrometry. Biochim. Biophys. Acta. Mol. Basis Dis. 1867 (11), 166210. 10.1016/j.bbadis.2021.166210 34246751

[B19] Li L. Zheng X. Zhou Q. Villanueva N. Nian W. Liu X. (2020). Metabolomics-based discovery of molecular signatures for triple negative Breast cancer in Asian female population. Sci. Rep. 10 (1), 370. 10.1038/s41598-019-57068-5 31941951 PMC6962155

[B20] Ma Y. Wang L. Jia R. (2020). The role of mitochondrial dynamics in human cancers. Am. J. Cancer Res. 10 (5), 1278–1293. 32509379 PMC7269774

[B21] McCartney A. Vignoli A. Biganzoli L. Love R. Tenori L. Luchinat C. (2018). Metabolomics in breast cancer: A decade in review. Cancer Treat. Rev. 67, 88–96. 10.1016/j.ctrv.2018.04.012 29775779

[B22] Narayanan R. Song X. Chen H. Zare R. N. (2020). Teflon spray ionization mass spectrometry. J. Am. Soc. Mass Spectrom. 31 (2), 234–239. 10.1021/jasms.9b00069 31939677

[B23] Nicholson J. K. Lindon J. C. (2008). Systems biology: Metabonomics. Nature 455 (7216), 1054–1056. 10.1038/4551054a 18948945

[B24] Ogretmen B. (2018). Sphingolipid metabolism in cancer signalling and therapy. Nat. Rev. Cancer 18 (1), 33–50. 10.1038/nrc.2017.96 29147025 PMC5818153

[B25] Pang Z. Chong J. Zhou G. de Lima Morais D. A. Chang L. Barrette M. (2021). MetaboAnalyst 5.0: Narrowing the gap between raw spectra and functional insights. Nucleic Acids Res. 49 (W1), W388–W396. 10.1093/nar/gkab382 34019663 PMC8265181

[B26] Quek L. E. van Geldermalsen M. Guan Y. F. Wahi K. Mayoh C. Balaban S. (2022). Glutamine addiction promotes glucose oxidation in triple-negative breast cancer. Oncogene 41 (34), 4066–4078. 10.1038/s41388-022-02408-5 35851845 PMC9391225

[B27] Scalise M. Pochini L. Console L. Losso M. A. Indiveri C. (2018). The human SLC1A5 (ASCT2) amino acid transporter: From function to structure and role in cell biology. Front. Cell Dev. Biol. 6, 96. 10.3389/fcell.2018.00096 30234109 PMC6131531

[B28] Schmid P. Rugo H. S. Adams S. Schneeweiss A. Barrios C. H. Iwata H. (2020). Atezolizumab plus nab-paclitaxel as first-line treatment for unresectable, locally advanced or metastatic triple-negative breast cancer (IMpassion130): Updated efficacy results from a randomised, double-blind, placebo-controlled, phase 3 trial. Lancet. Oncol. 21 (1), 44–59. 10.1016/S1470-2045(19)30689-8 31786121

[B29] Song X. Yang X. Narayanan R. Shankar V. Ethiraj S. Wang X. (2020). Oral squamous cell carcinoma diagnosed from saliva metabolic profiling. Proc. Natl. Acad. Sci. U. S. A. 117 (28), 16167–16173. 10.1073/pnas.2001395117 32601197 PMC7368296

[B30] Song X. Chen H. Zare R. N. (2018). Conductive polymer spray ionization mass spectrometry for biofluid analysis. Anal. Chem. 90 (21), 12878–12885. 10.1021/acs.analchem.8b03460 30247892

[B31] Takats Z. Strittmatter N. McKenzie J. S. (2017). Ambient mass spectrometry in cancer research. Adv. Cancer Res. 134, 231–256. 10.1016/bs.acr.2016.11.011 28110652

[B32] Tang Z. Kang B. Li C. Chen T. Zhang Z. (2019). GEPIA2: An enhanced web server for large-scale expression profiling and interactive analysis. Nucleic Acids Res. 47 (W1), W556–W560. 10.1093/nar/gkz430 31114875 PMC6602440

[B33] Thomas H. E. Zhang Y. Stefely J. A. Veiga S. R. Thomas G. Kozma S. C. (2018). Mitochondrial complex I activity is required for maximal autophagy. Cell Rep. 24 (9), 2404–2417. 10.1016/j.celrep.2018.07.101 30157433 PMC6298213

[B34] Toi M. Rahman M. A. Bando H. Chow L. W. (2005). Thymidine phosphorylase (platelet-derived endothelial-cell growth factor) in cancer biology and treatment. Lancet. Oncol. 6 (3), 158–166. 10.1016/S1470-2045(05)01766-3 15737832

[B35] Vander Heiden M. G. Cantley L. C. Thompson C. B. (2009). Understanding the Warburg effect: The metabolic requirements of cell proliferation. science 324 (5930), 1029–1033. 10.1126/science.1160809 19460998 PMC2849637

[B36] Xiao Y. Ma D. Yang Y. S. Yang F. Ding J. H. Gong Y. (2022). Comprehensive metabolomics expands precision medicine for triple-negative breast cancer. Cell Res. 32 (5), 477–490. 10.1038/s41422-022-00614-0 35105939 PMC9061756

[B37] Yang L. Wang Y. Cai H. Wang S. Shen Y. Ke C. (2020). Application of metabolomics in the diagnosis of breast cancer: A systematic review. J. Cancer 11 (9), 2540–2551. 10.7150/jca.37604 32201524 PMC7066003

[B38] Yang X. Song X. Yang X. Han W. Fu Y. Wang S. (2022). Big cohort metabolomic profiling of serum for oral squamous cell carcinoma screening and diagnosis. Nat. Sci. 2 (1), e20210071. 10.1002/ntls.20210071

[B39] Zhang B. Hu S. Baskin E. Patt A. Siddiqui J. K. Mathé E. A. (2018). RaMP: A comprehensive relational database of metabolomics pathways for pathway enrichment analysis of genes and metabolites. Metabolites 8 (1), 16. 10.3390/metabo8010016 29470400 PMC5876005

[B40] Zhou Y. Zhang J. Wang K. Han W. Wang X. Gao M. (2020). Quercetin overcomes colon cancer cells resistance to chemotherapy by inhibiting solute carrier family 1, member 5 transporter. Eur. J. Pharmacol. 881, 173185. 10.1016/j.ejphar.2020.173185 32422185

