# Supplementary material for: Screening and diagnosis of triple negative breast cancer based on rapid metabolic fingerprinting by conductive polymer spray ionization mass spectrometry and machine learning
Source: Front Cell Dev Biol. 2022 Dec 15;10:1075810. doi: 10.3389/fcell.2022.1075810 (PMC9798417; doi:10.3389/fcell.2022.1075810)
Supplement: Supplementary file 1 [file DataSheet1.pdf]

Supplementary Information

Screening and Diagnosis of Triple Negative Breast Cancer based on Rapid Metabolic Fingerprinting by Conductive Polymer Spray Ionization Mass Spectrometry and Machine Learning

Yaoyao Song[1,2#], Yan Zhang[1#], Songhai Xie[3], Xiaowei Song[3*]

[1] Department of General Surgery, the Sixth Medical Center of Chinese PLA General Hospital, Beijing, China, 100037.
[2] Department of Burn and Plastic Surgery, the Fourth Medical Center of Chinese PLA General Hospital, Beijing, China, 100048.

[3] Department of Chemistry, Fudan University, Shanghai, China, 200438.

***Correspondence:**
Xiaowei Song, songxw@fudan.edu.cn

# Authors contribute equally to this work.

Table of Contents

Contents	Legend	Page
Table S1	Demographics of the collected TNBC tissue and serum cases.	S3
Table S2	The significantly changed metabolites in TNBC tissue confirmed by DESI-MSI.	S4
Table S3	Selected metabolite markers and their weight coefficient in optimal Lasso model for complementary diagnosis.	S5
Table S4	The significantly changed metabolites discovered in the TNBC serum.	S6
Table S5	The significantly changed metabolites that were discovered both in tissue and serum.	S7
Table S6	Quantitative Estimation for the 21 metabolite markers' concentration in serum.	S8
Table S7	Metabolite markers and their weight coefficients in the Lasso classifier for serum TNBC screening.	S9
Table S8	Summary of altered metabolic pathways in tissues from TNBC group.	S10
Table S9	Summary of altered metabolic pathways in serum from TNBC group.	S11
Table S10	Transporter and enzymes prediction according to the top 5 enriched metabolism pathways.	S12
Table S11	Tentative identification of the metabolite ions.	S14
Figure S1	Average mass spectra of PNT and TNBC tissues under the positive full MS scan mode.	S17
Figure S2	The PLS-DA classification result for the TNBC and PNT cryosections.	S18
Figure S4	The quantitative comparison of remaining 8 serum metabolite markers.	S19

Figure S5	The dysregulated metabolism pathways highlighted in the tissue and serum metabolomics by the pathway analysis in the metaboanalyst.	S20

Table S1. Demographics of the collected TNBC tissue and serum cases.

**Characteristics**	**Tissue (n)**	**Serum (n)**
Race	Chinese	Chinese
Gender		
Female	43	242
Age	40-84	40-85
Metastasis		
Y	15	54
N	28	44
NA	0	144
Staging		
T1	9	38
T2	29	28
T3	5	38
T4	0	37
NA	0	131
Grading		
I	17	40
II	20	32
III	6	79
NA	0	91

NA: not available

Table S2. The significantly changed metabolites discovered in the TNBC tissues.

metabolite	FC*	FDR	metabolite	FC*	FDR
glutamate	0.00	1.05E-01	5-aminopentanamide	2.69	3.51E-06
asparagine	0.01	1.32E-01	oleoylcarnitine	2.72	1.34E-07
palmitic acid	0.04	1.46E-01	3-methylglutaconic acid	2.78	5.09E-04
hydroxytestosterone	0.04	6.78E-08	2-ketobutyric acid	2.93	3.55E-03
caprylic acid	0.05	4.09E-05	N-methylnicotinamide	2.94	1.51E-06
glycerophophocholine	0.07	7.46E-02	creatinine	2.96	1.74E-07
acetyllactosamine	0.07	2.88E-06	linoleyl carnitine	3.10	2.37E-05
lysoPC (6:0)	0.09	2.04E-07	ketodeoxycholic acid	3.16	1.58E-04
phosphorylcholine	0.12	1.96E-01	palmitoylcarnitine	3.19	1.48E-07
linoleic acid	0.13	4.12E-02	tryptophan	3.26	1.41E-04
S-adenosylhomocysteine	0.14	6.68E-08	phosphatidylserine	3.31	3.67E-06
DG (36:5)	0.17	2.13E-05	fucose	3.32	5.32E-04
N-acetyl--fucosyl-glucosamine	0.17	3.15E-06	phenylalanine	3.40	1.18E-04
N-methyllysine	0.18	4.69E-03	hypoxanthine	3.48	3.56E-04
oleic acid	0.18	1.54E-05	PC (16:0/18:1)	3.54	1.14E-06
desaminotyrosine	0.19	6.32E-04	pipecolic acid	3.59	2.41E-07
5-hydroxylysine	0.23	1.01E-03	N-acetyl glucosamine	3.62	6.57E-05
lysoPC (P-18:0)	0.24	7.18E-01	acetylcarnitine	3.78	6.07E-07
glycerylphosphorylethanolamine	0.27	1.96E-02	PE (22:1/15:0)	4.20	1.48E-07
3-methylcytidine	0.28	4.10E-02	PC (16:0/16:0)	4.25	8.27E-08
progesterone	0.33	1.76E-04	propenoyl carnitine	4.47	6.21E-04
hypotaurine	0.36	3.67E-05	threonine	4.53	3.56E-05
3-Hydroxyquinine	0.36	2.31E-05	tyrosine	5.24	1.17E-04
valine betaine	1.33	5.36E-01	N-acetyl spermidine	5.32	8.00E-03
dimethylarginine	1.78	1.95E-02	hexanoyl carnitine	5.33	7.63E-08
pentadecanoylcarnitine	2.09	8.05E-03	methyl-thioguanosine monophosphate	5.76	6.53E-05
decadienoic acid	2.24	2.79E-07	DG (40:0)	5.83	1.31E-07
adipic acid	2.30	4.44E-02	phosphoserine	6.68	5.87E-03
oxoguanine	2.35	3.45E-04	maltotriose	7.57	2.40E-07
asparaginylcysteine	2.39	1.09E-03	lactose	7.90	5.45E-03
glycerol 1-monostearate	2.45	9.21E-08	kynurenate	8.66	4.94E-04
succinoadenosine	2.45	3.19E-06	leucine	8.80	6.09E-02
taurine	2.45	8.86E-05	methionine	9.50	3.23E-04
N-galacturonyl-lysine	2.50	3.43E-06	butyrylcarnitine	10.82	2.20E-07
uridine	2.51	1.83E-07	stearoylcarnitine	11.33	1.34E-05
3-aminopropionaldehyde	2.54	1.49E-02	proline	14.72	3.70E-02

tryptamine	2.62	2.92E-01	linoelaidyl carnitine	15.93	3.39E-05
phosphorylserine	2.67	1.45E-04	citrulline	16.78	1.80E-04

*FC: fold change, triple negative breast cancer (TNBC) vs heathly donor (HD).

Table S3. Selected metabolite markers and their weight coefficient in optimal Lasso model for complementary diagnosis.

m/z	weight	metabolite	adduct ion
343.1685	-0.13051	DG(O-16:0/18:0)	$[M+K]^+$
118.0614	-0.08251	1-butylamine	$[M+2Na-H]^+$
244.0928	-0.06627	cytidine	$[M+H]^+$
112.0871	-0.04978	histamine	$[M+H]^+$
184.0727	-0.04359	phosphorylcholine	$[M+H]^+$
339.1923	-0.02504	hydroxylinolenic acid	$[M+2Na-H]^+$
357.1587	-0.02152	linoleic acid	$[M+2K-H]^+$
195.0022	-0.01417	glycerol 3-phosphate	$[M+Na]^+$
148.0604	-0.01239	glutamate	$[M+H]^+$
216.1202	-0.00603	propenoyl carnitine	$[M+H]^+$
147.0764	0.005117	glutamine	$[M+H]^+$
204.0627	0.005901	tyrosine	$[M+Na]^+$
295.2263	0.023645	3-hydroxypalmitic acid	$[M+Na]^+$
518.2994	0.026051	lysoPC(P-16:0)	$[M+K]^+$
254.1359	0.028625	butyryl carnitine	$[M+Na]^+$
259.1663	0.028915	pipecolic acid	$[2M+H]^+$
558.2941	0.036762	lysoPC(18:2)	$[M+K]^+$
188.1753	0.048524	N-acetyl spermidine	$[M+H]^+$
135.0026	0.052666	lactic acid	$[M+2Na-H]^+$
178.0587	0.075176	histidine	$[M+Na]^+$
126.1027	0.083991	N-methyl histamine	$[M+H]^+$
192.0552	0.117344	N-acetyl histamine	$[M+K]^+$

Table S4. The significantly changed metabolites discovered in the TNBC serum.

metabolites	FC*	FDR	metabolites	FC*	FDR
1-butylamine	0.02	1.93E-28	kynurenate	1.67	2.59E-10
2-heptenoic acid	0.66	1.39E-04	leucine/isoleucine	1.81	2.26E-10
2-ketobutyric acid	5.82	8.79E-36	linoleic acid	0.17	3.07E-09
3-aminopropionaldehyde	1.62	9.11E-10	palmitic acid	0.34	5.72E-04
3-hydroxyquinine	144	7.57E-25	lysoPC(18:2)	17.00	1.74E-44
3-methylcytidine	0.45	6.44E-11	lysoPC(P-18:0)	0.08	4.09E-12
3-methylglutaconic acid	10.65	4.58E-42	lysoPE(20:3)	0.85	2.86E-02
3-oxohexadecanoic acid	1.25	1.12E-02	methionine	1.60	7.43E-10
4-acetamido-2-aminobutanoic acid	0.01	1.64E-92	MG(18:0)	0.01	2.20E-03
8-oxoguanine	0.70	3.84E-05	N-methyl lysine	0.16	3.01E-15
acetylcarnitine	8.27	1.98E-29	N, N-diacetyl spermine	0.59	4.64E-06
adipic acid	0.02	5.24E-26	N-acetyl spermidine	6.44	1.25E-28
asparagine	0.08	2.39E-14	N-acetyl cadaverine	1.16	2.29E-02
aspartate	0.03	8.29E-13	N-acetyl cystathionine	4.48	2.94E-02
butyryl carnitine	10.11	2.18E-05	N-methyl nicotinamide	9.77	1.41E-13
caprylic acid	0.24	2.48E-17	oleic acid	65.31	1.98E-29
creatinine	2.89	4.39E-26	pentadecenoyl carnitine	2.23	1.61E-08
cysteic acid	0.22	1.71E-05	palmitoyl carnitine	1.01	1.26E-03
decadienoic acid	1.15	5.37E-03	PC (16:0/16:1)	4.04	2.59E-10
deoxyuridine	8.49	8.72E-13	PC (16:0/18:1)	1.87	2.84E-05
DG (18:1/22:0/0:0)	4.32	1.73E-03	PC (18:1/16:1)	1.94	5.58E-10
dimethylarginine	6.97	7.77E-19	phenylalanine	0.92	3.92E-02
fucose	1.86	1.70E-13	phosphate	0.51	9.67E-06
glutamate	0.16	4.14E-16	phosphorylcholine	0.24	5.72E-10
glycerol	3.66	3.79E-16	phosphoserine	2.99	1.11E-17
glycerol 1-monostearate	1.20	3.19E-03	proline	1.71	1.38E-09
glycerophophocholine	0.03	1.05E-18	propenoyl carnitine	5.24	5.80E-29
histidine	0.25	4.03E-04	S-adenosylhomocysteine	4.24	1.65E-02
homogentisic acid	0.17	4.68E-09	succinoadenosine	10.90	6.24E-22
hydroxylinoleic acid	1.07	4.43E-02	taurine	0.15	6.55E-20
hydroxyoleic acid	4.00	2.98E-03	taurocholic acid	2.24	2.29E-02
hydroxyprogesterone	0.05	3.53E-09	threonine	1.12	1.20E-03
hydroxytestosterone	0.05	9.35E-10	trimethyllysine	Inf	2.38E-02
hypoxanthine	0.26	1.38E-15	tryptamine	73.14	1.93E-05
inosine	0.13	7.23E-33	tryptophan	10.95	1.80E-41
ketodeoxycholic acid	1.08	2.50E-02	valine betaine	34.15	1.45E-05

ketoisovaleric acid	1.36	4.63E-02

*FC: fold change, triple negative breast cancer (TNBC) vs heathly donor (HD).

Table S5. The significantly changed metabolites that were discovered both in tissue and serum.

Metabolite	FC (tissue)	FDR (tissue)	FC (serum)	FDR (serum)	DESI-MSI Validation
2-Ketobutyric acid	2.62	2.20E-02	5.37	8.37E-36	Y
acetyl carnitine	2.96	1.31E-02	5.92	1.31E-13	Y
asparagine	0.12	2.14E-03	0.03	1.06E-06	Y
butyryl carnitine	5.34	1.07E-03	4.99	1.77E-02	Y
caprylic acid	0.06	9.63E-04	0.10	1.45E-06	Y
creatinine	3.16	3.10E-03	2.43	8.39E-12	Y
dimethylarginine	1.84	6.52E-03	3.56	5.99E-07	Y
fucose	3.49	3.97E-03	1.19	1.65E-02	N
glutamate	0.00	1.29E-02	0.09	7.26E-14	Y
glutamine	0.11	3.26E-02	0.33	1.50E-07	Y
glycerophophocholine	0.15	1.04E-03	0.01	2.88E-07	Y
kynurenate	4.41	3.73E-03	1.18	1.20E-01	Y
leucine	8.56	8.68E-03	1.28	1.82E-02	Y
linoleic acid	0.28	1.41E-02	0.07	1.18E-03	Y
linolenic acid	0.08	6.52E-03	0.12	1.05E-02	Y
methionine	4.41	3.73E-03	1.30	1.49E-02	Y
N-methylnicotinamide	3.17	3.80E-02	5.50	5.65E-04	Y
phosphorylcholine	0.17	1.48E-02	0.09	4.21E-07	Y
phosphoserine	5.95	1.89E-02	1.92	2.49E-02	Y
proline	13.66	9.75E-05	1.28	3.92E-02	Y
tryptamine	2.56	1.46E-02	45.95	0.00E+00	Y
tryptophan	2.87	3.07E-02	6.57	2.10E-17	Y
methylglutaconic acid	2.43	6.63E-03	7.40	1.33E-42	Y
DG(18:1/22:0)	3.27	1.82E-02	2.52	3.13E-02	Y
lysoPC(18:2)	0.44	5.17E-03	0.83	1.97E-02	Y
lysoPC(P-18:0)	0.29	1.04E-02	0.07	5.97E-10	Y

N-Methyl lysine	0.19	1.03E-03	0.07	4.48E-07	N
N-Acetyl spermidine	4.19	4.14E-02	4.76	3.88E-12	Y
PC(16:0/16:1)	3.23	2.04E-02	2.71	2.39E-03	Y
succinoadenosine	2.41	2.10E-02	9.48	4.39E-09	N

Table S6. Quantitative Estimation for the 21 metabolite markers' concentration in serum.

Metabolite	TNBC group (μM)		HD group (μM)		P value*
	Median (Min - Max)		Median (Min - Max)		
2-Ketobutyric acid	1.18	0.11-13.92	3.99	0.66-24.81	****
phosphoryl choline	0.01	0.00-0.52	0.02	0.00-1.10	ns
N-methyl nicotinamide	0.07	0.00-1.97	0.04	0.00-0.98	****
acetyl carnitine	3.67	0.43-14.14	4.80	0.26-37.98	**
phosphoserine	3.96	0.12-81.05	4.60	0.01-48.28	ns
creatinine	6.00	2.11-33.10	6.94	1.80-41.48	*
tryptamine	0.18	0.00-0.62	0.20	0.00-1.91	**
fucose	43.28	14.76-211.6	33.95	1.83-94.7	****
linolenic acid	61.31	0.23-618.3	41.67	0.27-528.4	**
linoleic acid	186.0	0.81-4263	85.5	0.63-1417	****
butyryl carnitine	0.032	0.011-0.26	0.046	0.001-0.5	****
kynurenate	6.23	0.35-32.00	3.78	0.18-11.57	***
glutamine	23.26	1.59-131.3	13.86	2.35-34.30	****
proline	16.92	0.74-55.13	16.15	0.17-103.1	ns
methionine	6.57	0.098-21.66	8.17	0.13-29.47	**
leucine/Isoleucine	12.72	0.01-44.15	14.66	0.34-62.63	*
tryptophan	77.74	0.78-13.63	43.21	1.82-226.3	****
glutamate	156.5	6.03-844.7	26.22	2.04-216.4	****
asparagine	27.48	1.80-93.57	3.46	0.01-17.76	****

caprylic acid	16.17	1.57-378.21	1.51	0.00-29.28	****
glycerophosphocholine	0.33	0.00-23.85	5.16	0.15-26.55	****

ns: non-significance; *P<0.05; ** P<0.01; *** P<0.001; **** P<0.0001
*Rank-sum test

Table S7. Metabolite markers and their weight coefficients in the Lasso classifier for serum TNBC screening.

No	metabolite	adduct	*m/z*	Weight
1	2-ketobutyric acid	$[M+Na]^+$	125.0207	-0.02366
2	creatinine	$[M+Na]^+$	136.0478	0.029274
3	N-methylnicotinamide	$[M+H]^+$	137.0706	0.003989
4	glutamate	$[M+H]^+$	148.0604	-0.00743
4’	glutamate	$[M+Na]^+$	170.0424	-0.01947
5	glutamine	$[M+Na]^+$	169.0581	0.017102
6	methionine	$[M+Na]^+$	172.0399	-0.01921
7	fucose	$[M+Na]^+$	187.0572	-0.04255
8	tryptamine	$[M+2Na-H]^+$	205.0676	0.030714
9	caprylic acid	$[M+2K-H]^+$	221.0339	-0.02231
10	acetyl carnitine	$[M+Na]^+$	226.1043	0.021278
11	tryptophan	$[M+Na]^+$	227.0788	-0.0938
11’	tryptophan	$[M+K]^+$	243.0533	0.024479
12	butyryl carnitine	$[M+Na]^+$	254.1359	0.004654
13	glycerophophocholine	$[M+H]^+$	258.1097	-0.01663
14	linoleic acid	$[M+H-H_2O]^+$	263.2363	-0.01832
15	linolenic acid	$[M+H+]^+$	279.2316	0.022817
	intercept	---	----	1.456686

Table S8. Summary of altered metabolic pathways in tissues from TNBC group.

pathway	$-log_{10}(P)$	FDR	Impact	Related metabolites
Phenylalanine, tyrosine and tryptophan biosynthesis	3.925	2.37E-01	1.00	phenylalanine; tyrosine
Linoleic acid metabolism	3.4538	3.32E-01	1.00	linoleate, phosphatidylcholine
Taurine and hypotaurine metabolism	7.2548	1.48E-02	0.71	cysteate, taurine, hypotaurine, taurocholate
Glycerophospholipid metabolism	8.6951	0.47E-02	0.56	Phosphatidylethanolamine, phosphatidylcholine, acyl-sn-glycero-3-phosphocholine, diacyl-sn-glycerol, choline phosphate, ethanolamine phosphate, phosphatidate, phosphatidylserine, sn-glycerol 3-phosphate, glycerophosphocholine, glycerophosphoethanolamine
D-Glutamine and D-glutamate metabolism	3.0877	3.48E-01	0.50	glutamate; glutamine; ketoglutarate
Histidine metabolism	6.3202	3.02E-02	0.43	glutamate; urocanate; histidine; methylhistamine; histamine; aspartate
Arginine biosynthesis	4.8745	1.07E-01	0.34	glutamate; arginine; citrulline; aspartate; ornithine; glutamine; ketoglutarate; fumarate
Cysteine and methionine metabolism	3.1347	3.48E-01	0.19	serine; methionine; S-adenosyl-homocysteine; cysteate; 2-oxobutanoate; phosphoserine
Pyrimidine metabolism	2.5297	4.94E-01	0.17	glutamine; uridine; cytidine; deoxyuridine; thymidine; uracil; alanine
Aminoacyl-tRNA biosynthesis	11.295	1.04E-03	0.00	asparagine; histidine; phenylalanine; arginine; glutamine; aspartate; serine; methionine; valine; alanine; cysteine; lysine; leucine/ isoleucine; threonine; tryptophan tyrosine; proline; glutamate
Valine, leucine and isoleucine biosynthesis	10.29	0.14E-02	0.00	threonine; leucine/isoleucine; 3-methyl-2-oxobutanoic acid; 2-oxobutanoate; 4-methyl-2-oxopentanoate; valine

Table S9. Summary of altered metabolic pathways in serum TNBC group.

Metabolic pathway	$-\log_{10}(P)$	FDR	Impact*	Related metabolites
Phenylalanine, tyrosine and tryptophan biosynthesis	7.0035	0.023178	1.0	phenylalanine; tyrosine;
Linoleic acid metabolism	3.3744	0.2876	1.0	linoleate; phosphatidylcholine
D-Glutamine and D-glutamate metabolism	3.0101	0.31847	0.5	glutamate; glutamine
Arginine and proline metabolism	3.5853	0.25881	0.3444	arginine; creatine; proline; glutamate; ornithine; phosphocreatine;
Glycine, serine and threonine metabolism	4.2452	0.17199	0.2916	serine; choline; betaine; glycerate; creatine; 2-oxobutanoate
Arginine biosynthesis	6.8091	0.023178	0.2538	glutamate; arginine; ornithine; glutamine; urea;
Aminoacyl-tRNA biosynthesis	12.665	0.000266	0.1667	asparagine; histidine; phenylalanine; arginine; glutamine; serine; valine; lysine; leucine; tryptophan; tyrosine; proline; glutamate
Pyrimidine metabolism	4.7243	0.12427	0.1651	glutamine; UMP; uridine; dihydrouracil; deoxyuridine
Sphingolipid metabolism	3.2736	0.2892	0.1582	sphinganine; sphingomyelin; serine; phytosphingosine
Lysine degradation	4.0816	0.17724	0.1409	lysine; 2-oxoadipate; pipecolate; hydroxylysine; N,N,N-trimethyl-lysine
Nitrogen metabolism	3.0101	0.31847	0	glutamate; glutamine

Valine, leucine, and isoleucine biosynthesis	7.0922	0.023178	0	leucine; 3-methyl-2-oxobutanoic acid; 2-oxobutanoate; valine

* The hypergeometric test was conducted in the enrichment analysis to evaluate the statistical significance of certain pathway, which yields the p value. The relative-betweenness centrality was used as the metric in topology analysis to evaluate the pathway impact, which ranges from zero to one.

Table S10. Involved transporters and enzymes prediction according to the enriched metabolism pathways by searching in KEGG and Reactome.

Pathway Name	Database	Enzyme	Prediction	P Value
Aminoacyl-tRNA biosynthesis	KEGG	FARSB	Down	0.0042
Central carbon metabolism in cancer	KEGG	SLC1A5	Up	0.0128
Central carbon metabolism in cancer	KEGG	LDHAL6A	Up	0.0540
Aminoacyl-tRNA biosynthesis	KEGG	EPRS	Down	0.0690
Aminoacyl-tRNA biosynthesis	KEGG	DARS2	Down	0.1122
Central carbon metabolism in cancer	KEGG	LDHB	Up	0.1122
Aminoacyl-tRNA biosynthesis	KEGG	WARS2	Down	0.1235
Aminoacyl-tRNA biosynthesis	KEGG	DARS	Down	0.1382
Aminoacyl-tRNA biosynthesis	KEGG	EARS2	Down	0.1382
Central carbon metabolism in cancer	KEGG	LDHA	Up	0.1541
Aminoacyl-tRNA biosynthesis	KEGG	WARS	Down	0.1685
Central carbon metabolism in cancer	KEGG	SLC16A3	Down	0.2515
Aminoacyl-tRNA biosynthesis	KEGG	NARS	Up	0.2846
Central carbon metabolism in cancer	KEGG	LDHC	Up	0.3221
Aminoacyl-tRNA biosynthesis	KEGG	YARS	Down	0.3253
Aminoacyl-tRNA biosynthesis	KEGG	FARSA	Down	0.4229
Aminoacyl-tRNA biosynthesis	KEGG	TARSL2	Down	0.4512
Aminoacyl-tRNA biosynthesis	KEGG	LARS2	Down	0.4570
Aminoacyl-tRNA biosynthesis	KEGG	QARS	Up	0.4987
Aminoacyl-tRNA biosynthesis	KEGG	HARS2	Down	0.5296
Aminoacyl-tRNA biosynthesis	KEGG	MARS2	Down	0.5811
Aminoacyl-tRNA biosynthesis	KEGG	LARS	Down	0.6144
Aminoacyl-tRNA biosynthesis	KEGG	HARS	Down	0.6279
Central carbon metabolism in cancer	KEGG	LDHAL6B	Up	0.6416
Aminoacyl-tRNA biosynthesis	KEGG	YARS2	Down	0.6416
Aminoacyl-tRNA biosynthesis	KEGG	NARS2	Up	0.6554
Aminoacyl-tRNA biosynthesis	KEGG	MARS	Down	0.7919
Aminoacyl-tRNA biosynthesis	KEGG	FARS2	Down	0.7992
Central carbon metabolism in cancer	KEGG	SLC7A5	Down	0.9045
Aminoacyl-tRNA biosynthesis	KEGG	PARS2	Down	0.9962

Table S10. (continued)

Pathway Name	Database	Enzyme	Prediction	P Value
Transport of vitamins, nucleosides, and related molecules	reactome	SLCO1B3	Down	0.0188
Nucleotide salvage	reactome	TYMP	Down	0.0305
Transport of vitamins, nucleosides, and related molecules	reactome	PDZD11	Up	0.0408
Transport of vitamins, nucleosides, and related molecules	reactome	SLCO1B1	Down	0.0464
Glycerophospholipid biosynthesis	reactome	PISD	Down	0.0499
Glycerophospholipid biosynthesis	reactome	CHKB	Up	0.0531
Nucleotide salvage	reactome	TK1	Up	0.0661
Nucleotide salvage	reactome	HPRT1	Down	0.0705
Glycerophospholipid biosynthesis	reactome	PLA2G15	Up	0.1018
Nucleotide salvage	reactome	CDA	Up	0.1059
Transport of vitamins, nucleosides, and related molecules	reactome	SLC29A2	Up	0.1212
Nucleotide salvage	reactome	ADA	Up	0.2475
SLC transporter disorders	reactome	SLC29A3	Up	0.2475
Transport of vitamins, nucleosides, and related molecules	reactome	SLCO1A2	Down	0.2909
Nucleotide salvage	reactome	PNP	Up	0.3159
Glycerophospholipid biosynthesis	reactome	ETNK1	Up	0.4011
Glycerophospholipid biosynthesis	reactome	ETNK2	Up	0.5811
Transport of vitamins, nucleosides, and related molecules	reactome	SLC5A6	Up	0.6010
Nucleotide salvage	reactome	TK2	Up	0.6904
Glycerophospholipid biosynthesis	reactome	CHKA	Up	0.7406
Nucleotide salvage	reactome	UPP1	Up	0.7624
Nucleotide salvage	reactome	UPP2	Up	0.8099
Transport of vitamins, nucleosides, and related molecules	reactome	ALB	Down	0.8440
Glycerophospholipid biosynthesis	reactome	PEMT	Down	0.8969
Transport of vitamins, nucleosides, and related molecules	reactome	SLC29A1	Up	0.9197
Glycerophospholipid biosynthesis	reactome	TMEM86B	Down	0.9809

Table S11. Tentative annotation of the metabolite ions.

metabolite	adduct ion	Exp. *m/z*	Theo. *m/z*	Formula	RE (ppm)*
12-ketodeoxycholic acid	$[M+Na]^+$	413.2652	413.2662	$C_{24}H_{38}O_4$	-2.51
1-butylamine	$[M+2Na-H]^+$	118.0604	118.0603	$C_4H_{11}N$	0.36
2-heptenoic acid	$[M+Na]^+$	151.0726	151.0726	$C_7H_{12}O_2$	0.00
2-ketobutyric acid	$[M+Na]^+$	125.0207	125.0206	$C_4H_6O_3$	0.00
3-aminopropionaldehyde	$[M+Na]^+$	96.04183	96.0420	C_3H_7NO	-1.82
3-hydroxyquinine	$[M+H]^+$	341.1845	341.1859	$C_{20}H_{24}N_2O_3$	-4.39
3-methylcytidine	$[M+Na]^+$	280.0916	280.0903	$C_{10}H_{15}N_3O_5$	4.23
3-methylglutaconic acid	$[M+Na]^+$	167.0312	167.0315	$C_6H_8O_4$	-1.94
3-oxohexadecanoic acid	$[M+K]^+$	309.1826	309.1826	$C_{16}H_{30}O_3$	-0.08
5-aminopentanamide	$[M+Na]^+$	139.0839	139.0841	$C_5H_{12}N_2O$	-1.68
5-hydroxylysine	$[M+H-H_2O]^+$	145.0971	145.0977	$C_6H_{14}N_2O_3$	-4.42
8-oxoguanine	$[M+NH_4]^+$	183.0625	183.0625	$C_7H_{12}O_4$	-0.03
acetylcarnitine	$[M+Na]^+$	226.1043	226.1049	$C_9H_{17}NO_4$	-2.98
adipic acid	$[M+Na]^+$	169.0469	169.0471	$C_6H_{10}O_4$	-1.47
asparagine	$[M+H]^+$	133.0608	133.0608	$C_4H_8N_2O_3$	0.00
asparaginylcysteine	$[M+H]^+$	236.0710	236.0700	$C_7H_{13}N_3O_4S$	4.34
aspartate	$[M+2Na-H]^+$	178.0086	178.0086	$C_4H_7NO_4$	0.00
butyrylcarnitine	$[M+H]^+$	232.1537	232.1543	$C_{11}H_{21}NO_4$	-2.64
caprylic acid	$[M+2K-H]^+$	221.0339	221.0340	$C_8H_{16}O_2$	-0.76
citrulline	$[M+Na]^+$	198.0849	198.0849	$C_6H_{13}N_3O_3$	0.03
creatinine	$[M+Na]^+$	136.0478	136.0481	$C_4H_7N_3O$	-1.95
cysteic acid	$[M+K]^+$	207.9679	207.9676	$C_3H_7NO_5S$	1.24
decadienoic acid	$[M+Na]^+$	191.1038	191.1042	$C_{10}H_{16}O_2$	-2.60
deoxyuridine	$[M+H-H_2O]^+$	211.0723	211.0713	$C_9H_{12}N_2O_5$	4.74
desaminotyrosine	$[M+2Na-H]^+$	211.0332	211.0341	$C_9H_{10}O_3$	-4.37
DG(14:1/22:4)	$[M+K]^+$	339.1349	339.1357	$C_{19}H_{24}O_3$	-2.45
DG(18:0/22:0)	$[M+2K-H]^+$	757.5527	757.5509	$C_{43}H_{84}O_5$	2.32
DG(18:1/22:0)	$[M+2K-H]^+$	755.5372	755.5352	$C_{43}H_{82}O_5$	2.56
dimethylarginine	$[M+H]^+$	203.1498	203.1502	$C_8H_{18}N_4O_2$	-2.01
fucose	$[M+Na]^+$	187.0572	187.0576	$C_6H_{12}O_5$	-2.60
glutamate	$[M+H]^+$	148.0604	148.0604	$C_5H_9NO_4$	0.00
glycerol	$[M+K]^+$	131.0101	131.0105	$C_3H_8O_3$	-2.72
glycerol 1-monostearate	$[M+Na]^+$	381.2969	381.2975	$C_{21}H_{42}O_4$	-1.71

glycerophophocholine	$[M+H]^+$	258.1097	258.1101	$C_8H_{20}NO_6P$	-1.47
glycerylphosphorylethanolamine	$[M+K]^+$	254.0185	254.0190	$C_5H_{14}NO_6P$	-2.04
hexanoyl carnitine	$[M+H]^+$	260.1854	260.1856	$C_{13}H_{25}NO_4$	-0.95

Table S11. (continued)

metabolite	adduct ion	Exp. *m/z*	Theo. *m/z*	Formula	RE (ppm)
histidine	$[M+Na]^+$	178.0587	178.0587	$C_6H_9N_3O_2$	-0.19
homogentisic acid	$[M+H]^+$	169.0490	169.0495	$C_8H_8O_4$	-3.16
hydroxylinoleic acid	$[M+K]^+$	335.1976	335.1983	$C_{18}H_{32}O_3$	-2.25
hydroxyoleic acid	$[M+K]^+$	337.2135	337.2139	$C_{18}H_{34}O_3$	-1.31
hydroxyprogesterone	$[M+2K-H]^+$	407.1397	407.1385	$C_{21}H_{30}O_3$	2.88
hydroxytestosterone	$[M+2K-H]^+$	381.1242	381.1229	$C_{19}H_{28}O_3$	3.48
hypotaurine	$[M+K]^+$	147.9833	147.9829	$C_2H_7NO_2S$	2.32
hypoxanthine	$[M+K]^+$	175.0011	175.0017	$C_5H_4N_4O$	-3.05
inosine	$[M+Na]^+$	291.0691	291.0699	$C_{10}H_{12}N_4O_5$	-3.01
ketoisovaleric acid	$[M+Na]^+$	139.0363	139.0366	$C_5H_8O_3$	-2.15
kynurenate	$[M+H-H_2O]^+$	172.0399	172.0399	$C_{10}H_7NO_3$	0.00
lactose	$[M+2Na-H]^+$	387.0866	387.0874	$C_{12}H_{22}O_{11}$	-2.12
leucine/isoleucine	$[M+2Na-H]^+$	176.0654	176.0660	$C_6H_{13}NO_2$	-2.52
linoelaidyl carnitine	$[M+Na]^+$	446.3241	446.3241	$C_{25}H_{45}NO_4$	0.00
linoleic acid	$[M+K]^+$	319.2024	319.2034	$C_{18}H_{32}O_2$	-3.00
linoleyl carnitine	$[M+H]^+$	424.3414	424.3421	$C_{25}H_{45}NO_4$	-1.69
lysoPC(18:2)	$[M+Na]^+$	542.3203	542.3217	$C_{26}H_{50}NO_7P$	-2.51
lysoPC(6:0)	$[M+2K-H]^+$	432.0962	432.0950	$C_{14}H_{30}NO_7P$	2.62
lysoPC(P-18:0)	$[M+K]^+$	546.3303	546.3320	$C_{26}H_{54}NO_6P$	-3.12
lysoPE(20:3)	$[M+Na]^+$	526.2898	526.2904	$C_{25}H_{46}NO_7P$	-1.08
maltotriose	$[M+Na]^+$	527.1583	527.1583	$C_{18}H_{32}O_{16}$	0.00
methionine	$[M+Na]^+$	172.0403	172.0403	$C_5H_{11}NO_2S$	0.10
MG(18:0)	$[M+2Na-H]^+$	403.2811	403.2795	$C_{21}H_{42}O_4$	4.14
N-methyl lysine	$[M+2K-H]^+$	237.0402	237.0402	$C_7H_{16}N_2O_2$	-0.11
N, N-diacetyl spermine	$[M+2Na-H]^+$	331.2085	331.2080	$C_{14}H_{30}N_4O_2$	1.60
N-acetyl spermidine	$[M+H]^+$	188.1753	188.1757	$C_9H_{21}N_3O$	-1.90
N-acetyl cadaverine	$[M+K]^+$	183.0887	183.0895	$C_7H_{16}N_2O$	-3.97
N-acetyl glucosamine	$[M+Na]^+$	244.0786	244.0792	$C_8H_{15}NO_6$	-2.22
N-acetyl cystathionine	$[M+H]^+$	265.0853	265.0852	$C_9H_{16}N_2O_5S$	0.26
N-acetyl lactosamine	$[M+Na]^+$	406.1323	406.1320	$C_{14}H_{25}NO_{11}$	0.82
N-methyl nicotinamide	$[M+H]^+$	137.0706	137.0709	$C_7H_8N_2O$	-2.66
oleic acid	$[M+K]^+$	321.2184	321.2190	$C_{18}H_{34}O_2$	-2.10
oleoyl carnitine	$[M+H]^+$	426.3568	426.3578	$C_{25}H_{47}NO_4$	-2.38

pentadecenoyl carnitine	$[M+Na]^+$	408.3076	408.3084	$C_{22}H_{43}NO_4$	-1.94
palmitic acid	$[M+Na]^+$	279.2302	279.2295	$C_{16}H_{32}O_2$	2.56
palmitoyl carnitine	$[M+H]^+$	400.3415	400.3421	$C_{23}H_{45}NO_4$	-1.45

Table S11. (continued)

metabolite	adduct ion	Exp. *m/z*	Theo. *m/z*	Formula	RE (ppm)
PC(16:0/16:0)	$[M+Na]^+$	756.5506	756.5514	$C_{40}H_{80}NO_8P$	-1.02
PC(16:0/16:1)	$[M+Na]^+$	754.5347	754.5357	$C_{40}H_{78}NO_8P$	-1.37
PC(16:0/18:1)	$[M+Na]^+$	782.5646	782.5670	$C_{42}H_{82}NO8P$	-3.09
PC(18:1/16:1)	$[M+Na]^+$	780.5488	780.5514	$C_{42}H_{80}NO_8P$	-3.30
phenylalanine	$[M+Na]^+$	188.0678	188.0682	$C_9H_{11}NO_2$	-2.30
phosphatidylserine	$[M+H-H_2O]^+$	368.1128	368.1111	$C_{13}H_{24}NO_{10}P$	4.64
phosphorylcholine	$[M+H]^+$	184.0737	184.0739	$C_5H_{15}NO_4P$	-1.02
phosphoserine	$[M+Na]^+$	207.9979	207.9979	$C_3H_8NO_6P$	0.00
progesterone	$[M+2K-H]^+$	391.1434	391.1436	$C_{21}H_{30}O_2$	-0.66
proline	$[M+Na]^+$	138.0523	138.0526	$C_5H_9NO_2$	-1.56
propenoylcarnitine	$[M+H]^+$	216.1231	216.1230	$C_{10}H_{17}NO_4$	0.38
S-adenosylhomocysteine	$[M+2Na-H]^+$	429.0914	429.0928	$C_{14}H_{20}N_6O_5S$	-3.16
stearoyl carnitine	$[M+H]^+$	428.3723	428.3734	$C_{25}H_{49}NO_4$	-2.66
succinoadenosine	$[M+Na]^+$	406.0963	406.0969	$C_{14}H_{17}N_5O_8$	-1.49
taurine	$[M+Na]^+$	148.0035	148.0039	$C_2H_7NO_3S$	-2.86
taurocholic acid	$[M+2Na-H]^+$	560.2616	560.2632	$C_{26}H_{45}NO_7S$	-2.74
threonine	$[M+Na]^+$	142.0471	142.0475	$C_4H_9NO_3$	-2.41
trimethyllysine	$[M+Na]^+$	211.1416	211.1417	$C_9H_{20}N_2O_2$	-0.47
tryptamine	$[M+2Na-H]^+$	205.0716	205.0712	$C_{10}H_{12}N_2$	2.07
tryptophan	$[M+Na]^+$	227.0786	227.0791	$C_{11}H_{12}N_2O_2$	-2.11
tyrosine	$[M+Na]^+$	204.0627	204.0631	$C_9H_{11}NO_3$	-2.21
uridine	$[M+H]^+$	245.0780	245.0768	$C_9H_{12}N_2O_6$	4.77
valine betaine	$[M+K]^+$	198.0885	198.0891	$C_8H_{18}NO_2$	-3.00

*RE: Relative error of the measured m/z value compared to the theoretical value.

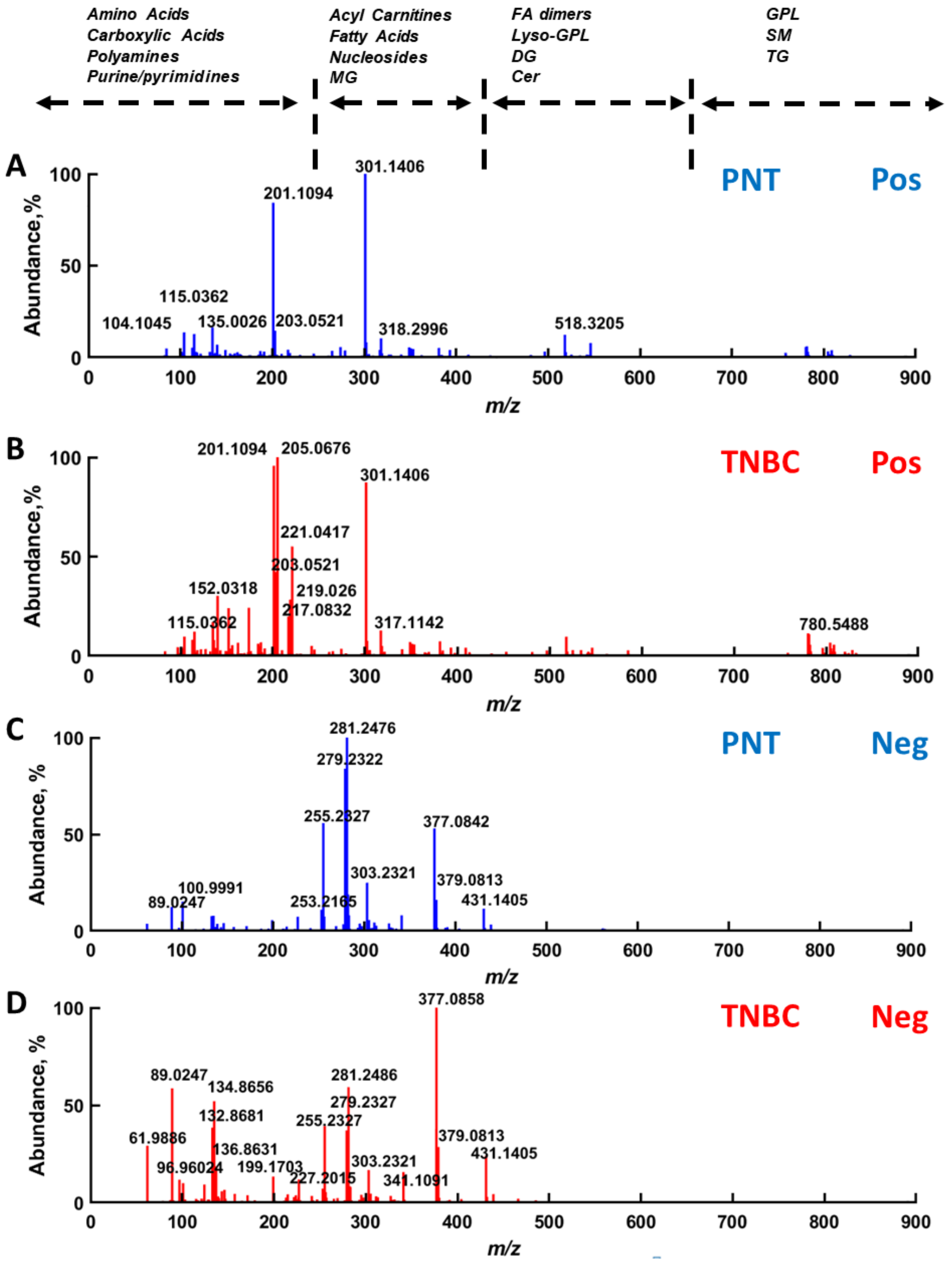

Figure S1. Mass spectral profiles acquired from the PNT (A, C) and TNBC (B, D) tissues under both the positive and negative full scan mode. The PNT and TNBC mass spectra were displayed in blue and red color, respectively.

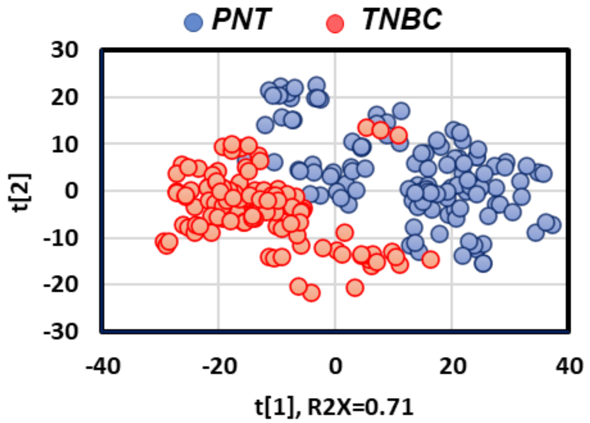

Figure S2. The PLS-DA classification result for the TNBCs (red points) and PNTs (blue points). (40 pairs with 3 extraction sample points from each tissue section).

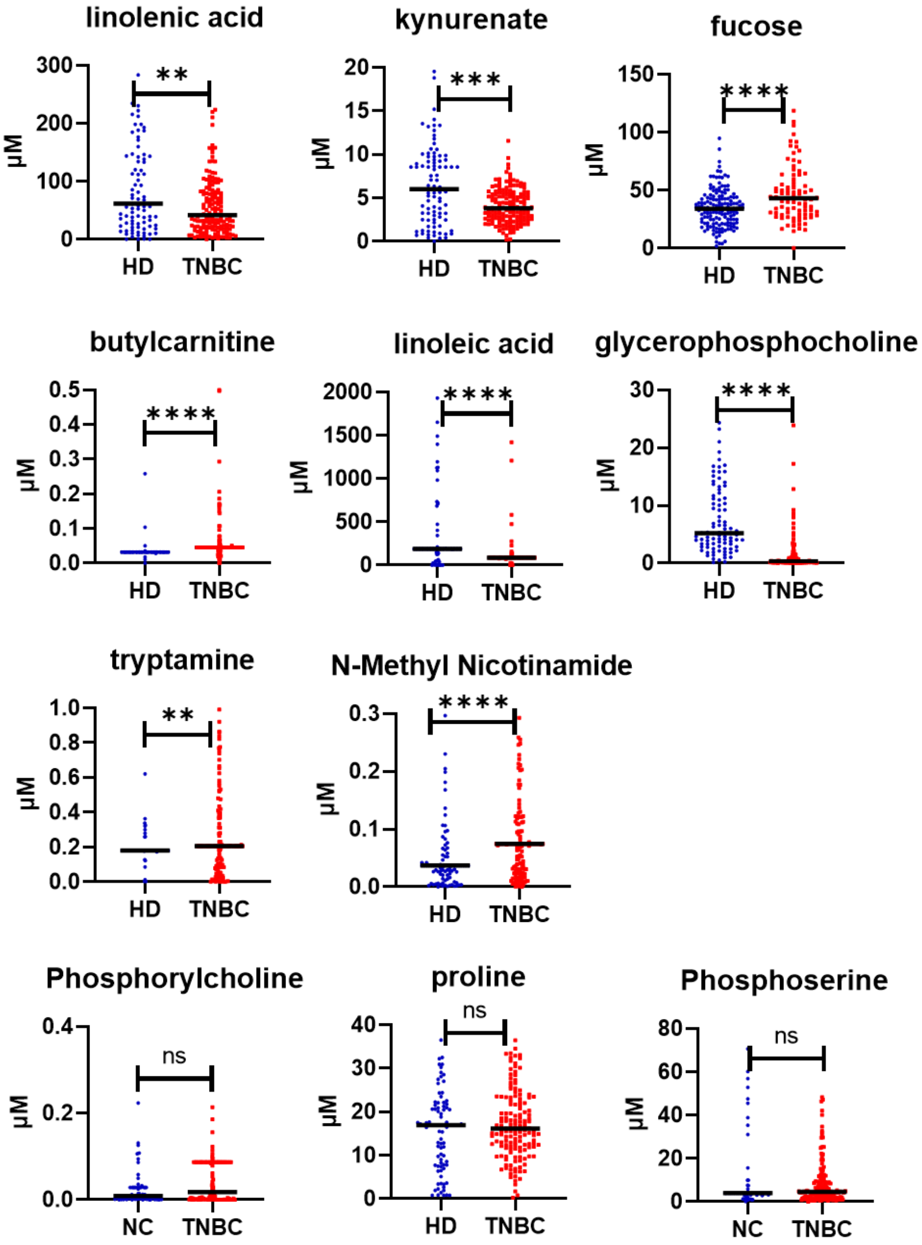

Figure S3. The quantitative comparison of remaining 11 serum metabolite markers. ns no statistical significance; *P<0.05; **P<0.01; ***P<0.001; ****P<0.0001;

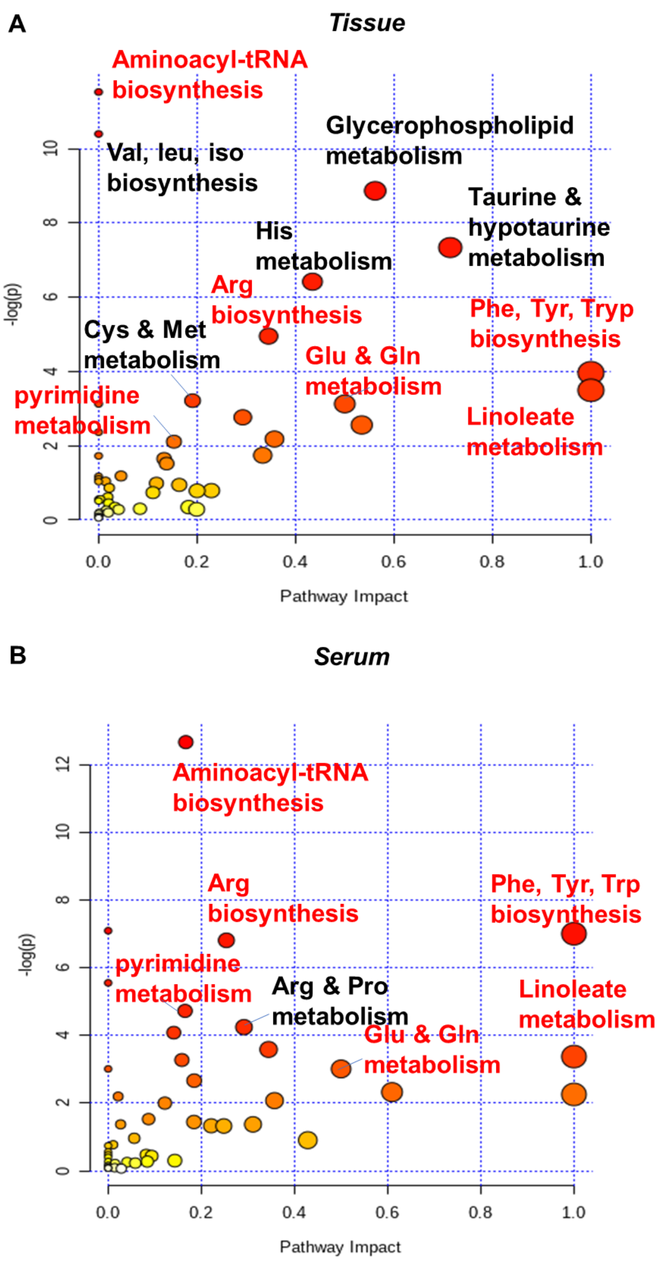

Figure S4. The dysregulated metabolism pathways highlighted in the tissue and serum metabolomics by the pathway analysis in the metaboanalyst.
Annotations: Arg, arginine; Phe, phenylalanine; Tyr, tyrosine; Glu, glutamate; Gln, glutamine; Pro, proline; His, histidine; Cys, cysteine; Met, methionine.
